# Supplementary material for: Mice mucosal leishmaniasis model shown high parasite load, increased cytotoxicity and impaired IL-10+ T cell response
Source: Front Immunol. 2025 Aug 4;16:1621781. doi: 10.3389/fimmu.2025.1621781 (PMC12358393; doi:10.3389/fimmu.2025.1621781)
Supplement: Supplementary file 1 [file Image1.pdf]

Supplementary Material

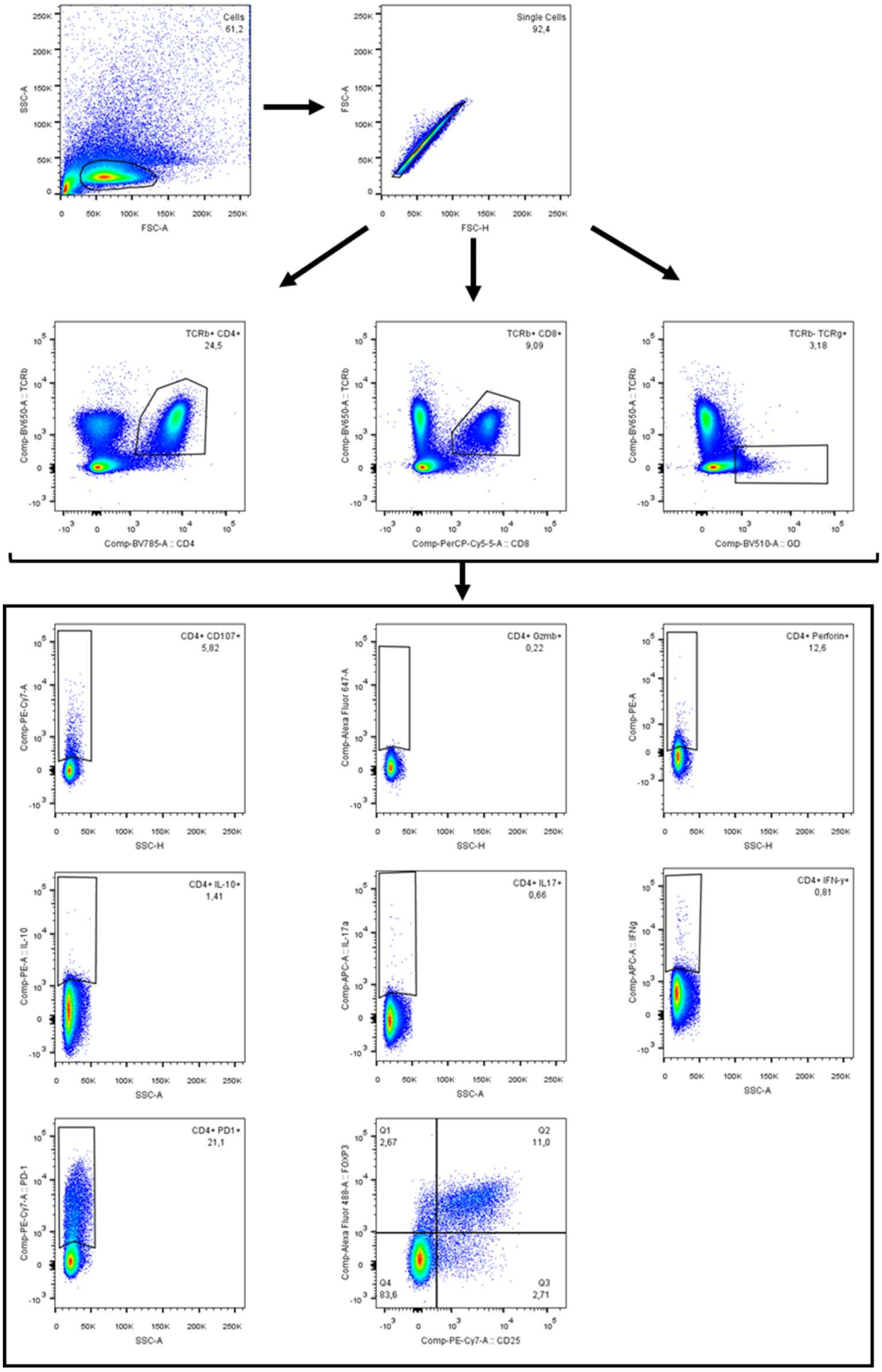

Figure S1. Gating strategy. The figure shows the gating strategy used to find and analyze CD4^{+} T, CD8^{+} T and Tγδ^{+} T cells. Within each population, a gate was performed to highlight cells

expressing the molecules CD107a, Perforin, Granzyme B, IL10, IL17 and IFN-γ (except Tγδ⁺) and PD-1. In $CD4^+$ T cells, the concomitant expression of CD25 and FoxP3 was also verified.

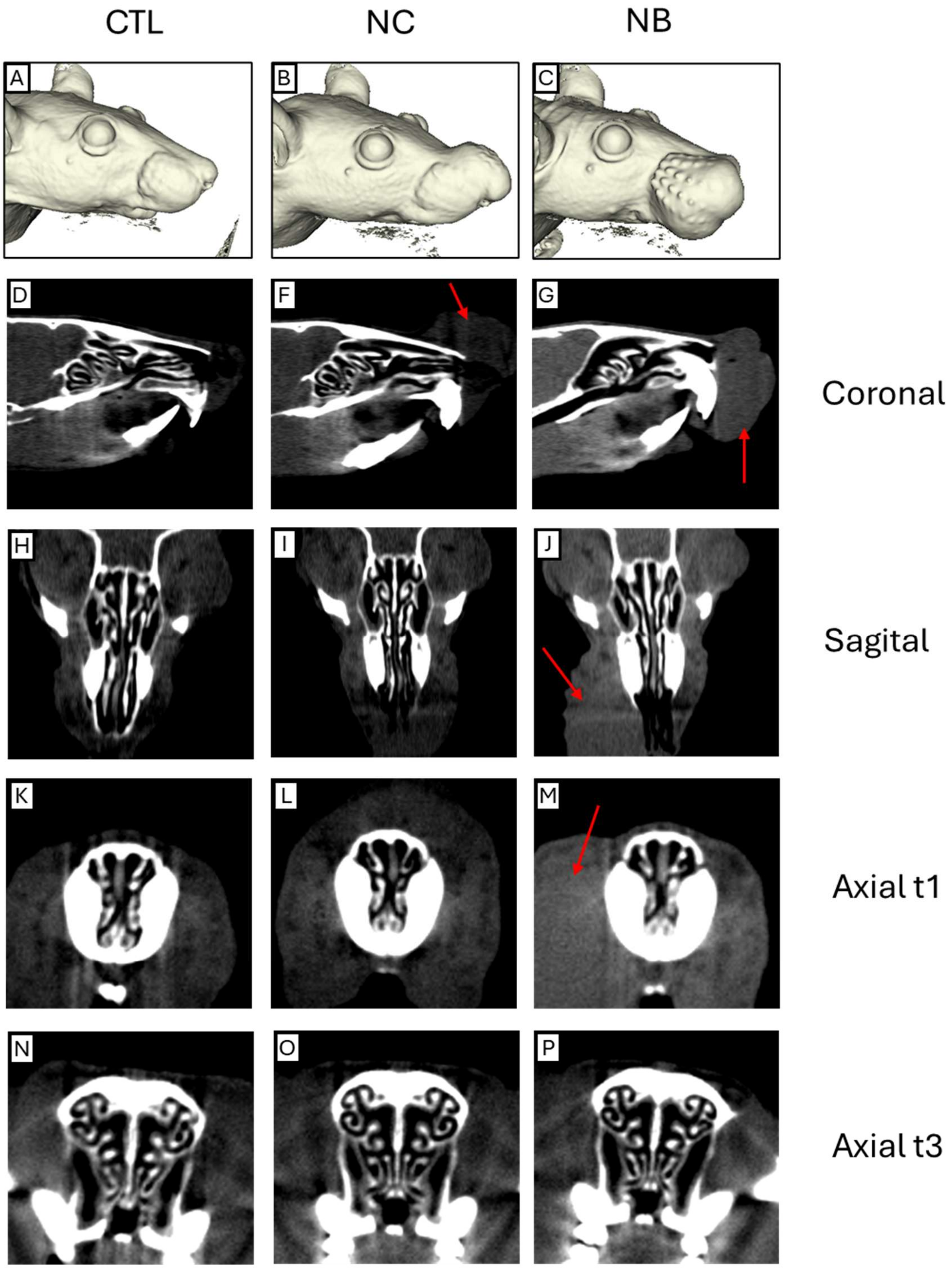

Figure S2. Small animal computed tomography (microCT). The complete three-dimensional reconstructions of the mice (first row). The microCT of the Control (CTL) mock PBS injection in the three sites, Nose Cutaneous (NC) and Nasobasal (NB) groups is also shown, under different

section planes: Coronal, Sagittal and Axial at t1 and t3. The red arrows indicate the region of edema. The group NC showed an edema in the nostril region that can progress to the upper part of the snout (second column. While NB infection mode led to a progressive swelling of premaxillary region (third column). No nasal cavity formation was observed, in any of the segments and infection groups.

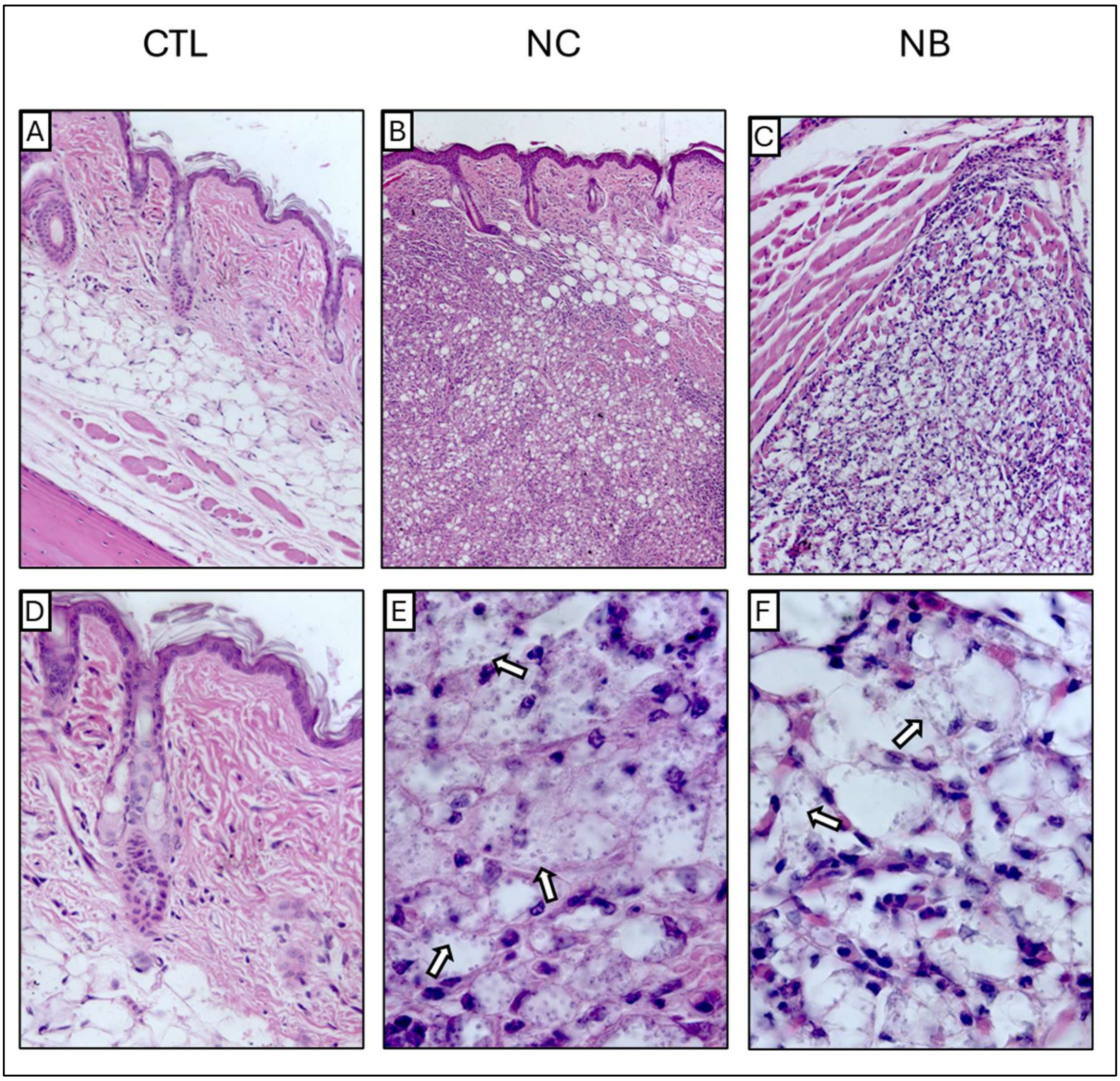

Figure S3: Histopathological Sections of the Epidermal Region of the Nose. Histopathological appearance of the nasal skin tissue from the Control (CTL) mock PBS injection in the three sites, Cutaneous Nose (NC) and Nasobasal (NB) groups, 12 weeks after the start of the experiment, stained with hematoxylin and eosin (H&E). The animals were infected with $2x10^6$ parasites**.** **(A, D)** appearance of the dermis of control animals. **(B, E)** NC group showing inflammatory infiltrate containing infected macrophages, below the epithelium. **(C, F)** NB group

showing intense inflammatory infiltrate with parasitized macrophages. Arrows shows examples of macrophages bearing amastigotes. Magnification: (A) 200x; (B) 100x; (C) 100x; (D); 400x; (E) 1000x and (F) 1000x.

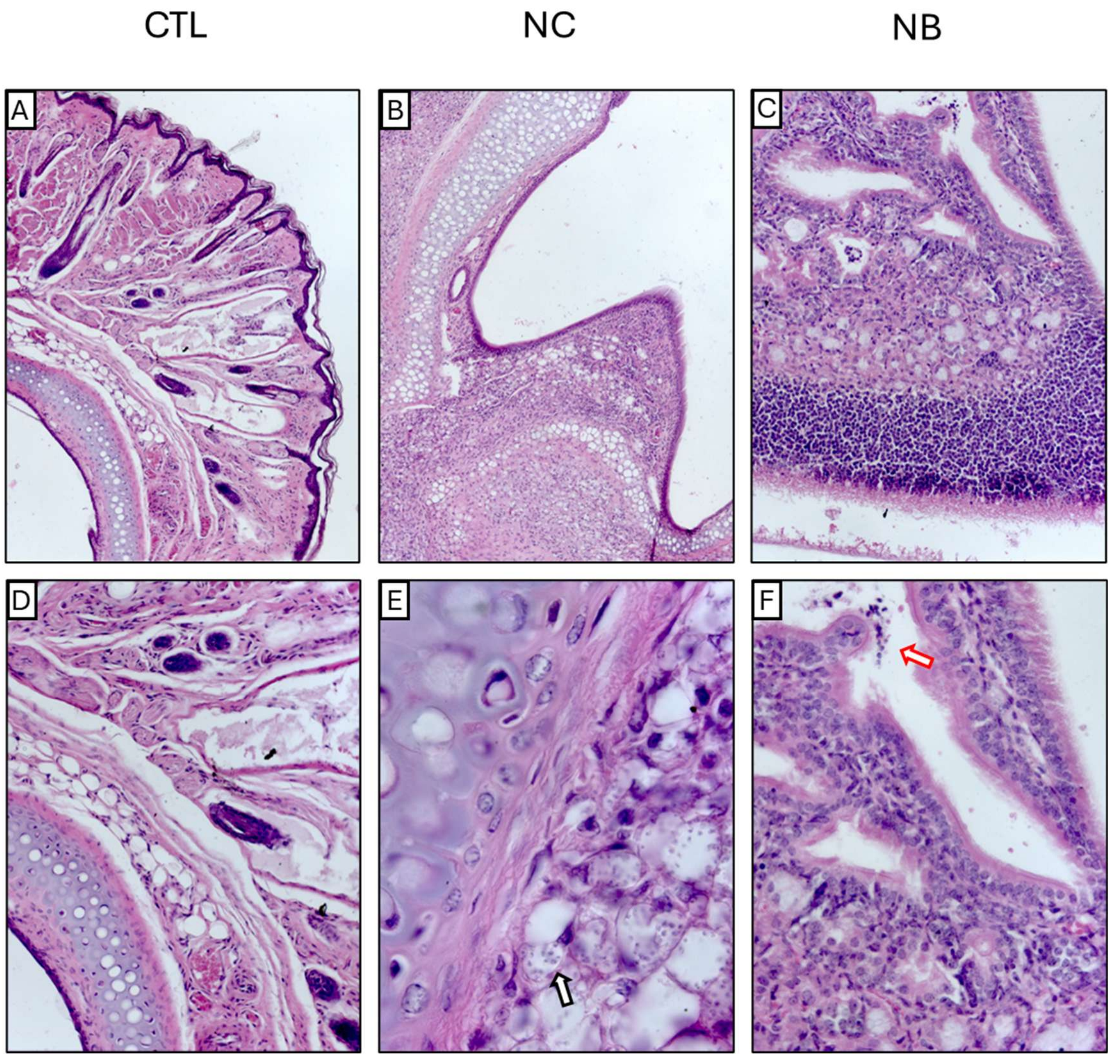

Figure S4: Histopathological Sections of the Initial Internal Region (T1) of the Nasal Mucosa. Histopathological appearance of the nasal tissue from the Control (CTL) mock PBS injection in the three sites, Cutaneous Nose (NC) and Nasobasal (NB) groups, 12 weeks after the start of the experiment, stained with hematoxylin and eosin (H&E). The animals were infected with $2x10^6$ parasites. **(A, D)** appearance of the dermis and squamous epithelium of control animals. **(B, E)** NC group showing inflammatory infiltrate containing infected macrophages, close to the cartilaginous regions. **(C, F)** NB group, demonstrating stress and structural disorganization of the tissue accompanied by cell detachment. White arrows show examples of macrophages

bearing amastigotes, red arrows show example of cell detachment. Magnification: (A) 100x; (B) 100x; (C) 100x; (D); 200x; (E) 1000x and (F) 200x.

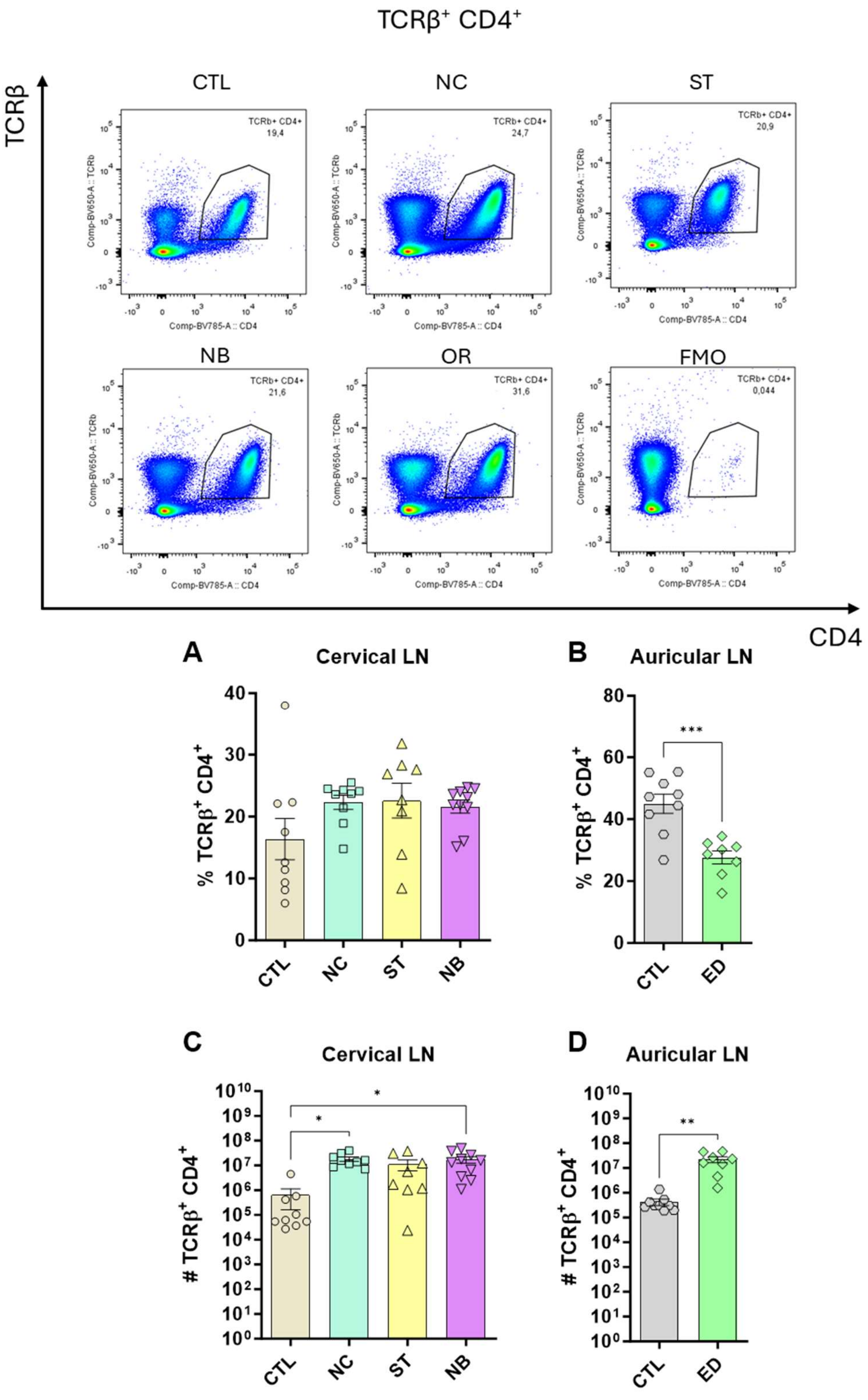

Figure S5. Profile of TCRβ⁺ CD4⁺ Cells. The figure shows the representatives and graphs with the percentage of cells in the nasal and ear sites (A) and (B) respectively. In (C) and (D) data on the total number of cells are represented. Data accumulative of two independent experiments.

Statistics: plot with Standard Error of The Mean (SEM), t-test was used for all groups and samples

* p<0,05, **<0,005., 3-6 animals per group in each.

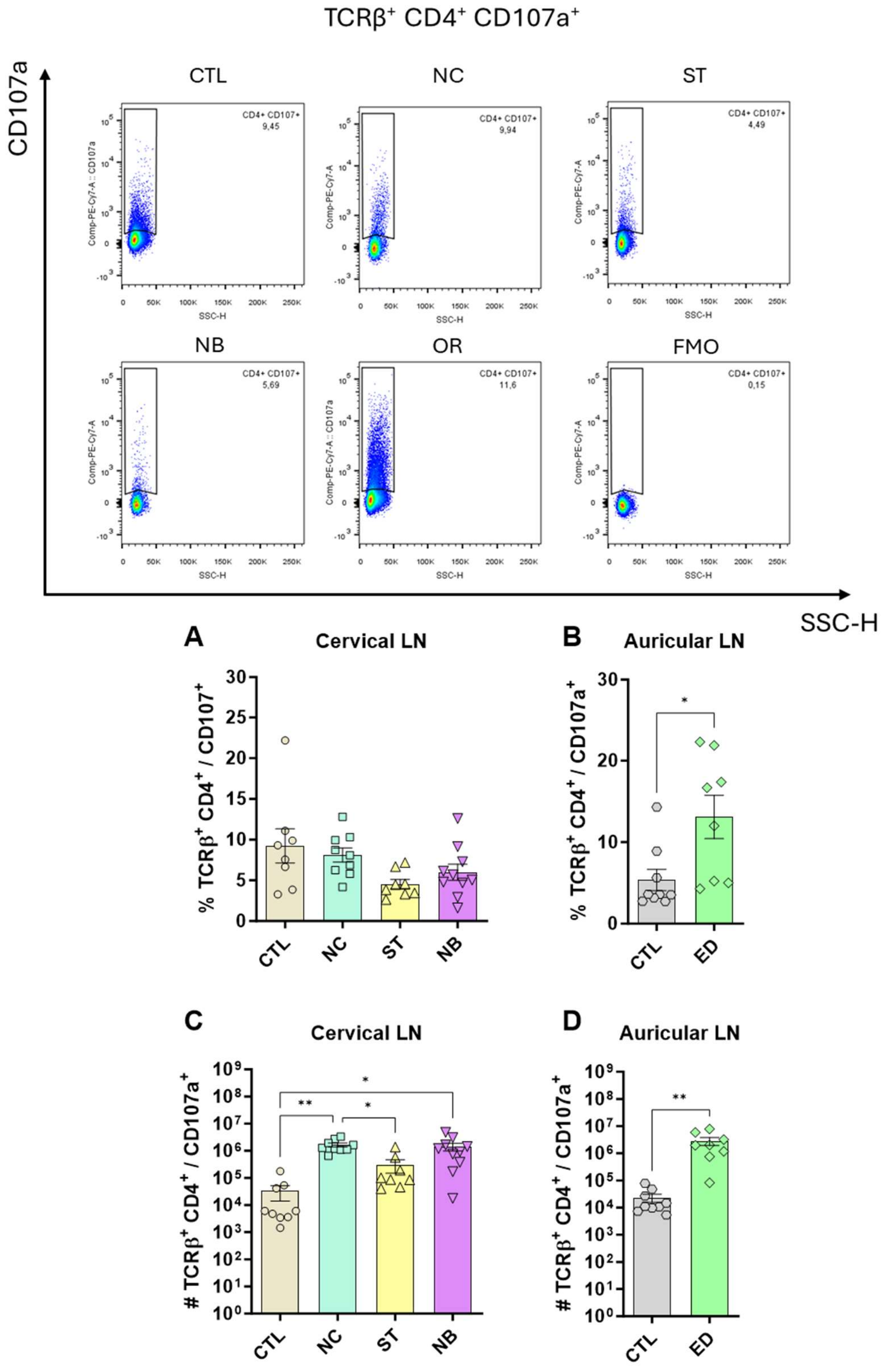

Figure S6. Profile of TCRβ$^+$ CD4$^+$ CD107a$^+$ Cells. The figure shows the representatives and graphs with the percentage of cells in the nasal and ear sites (A) and (B) respectively. In (C) and (D) data on the total number of cells are represented. Data accumulative of two independent experiments. Statistics: plot with Standard Error of The Mean (SEM), t-test was used for all groups and samples * $p<0{,}05$, **<0,005., 3-6 animals per group in each.

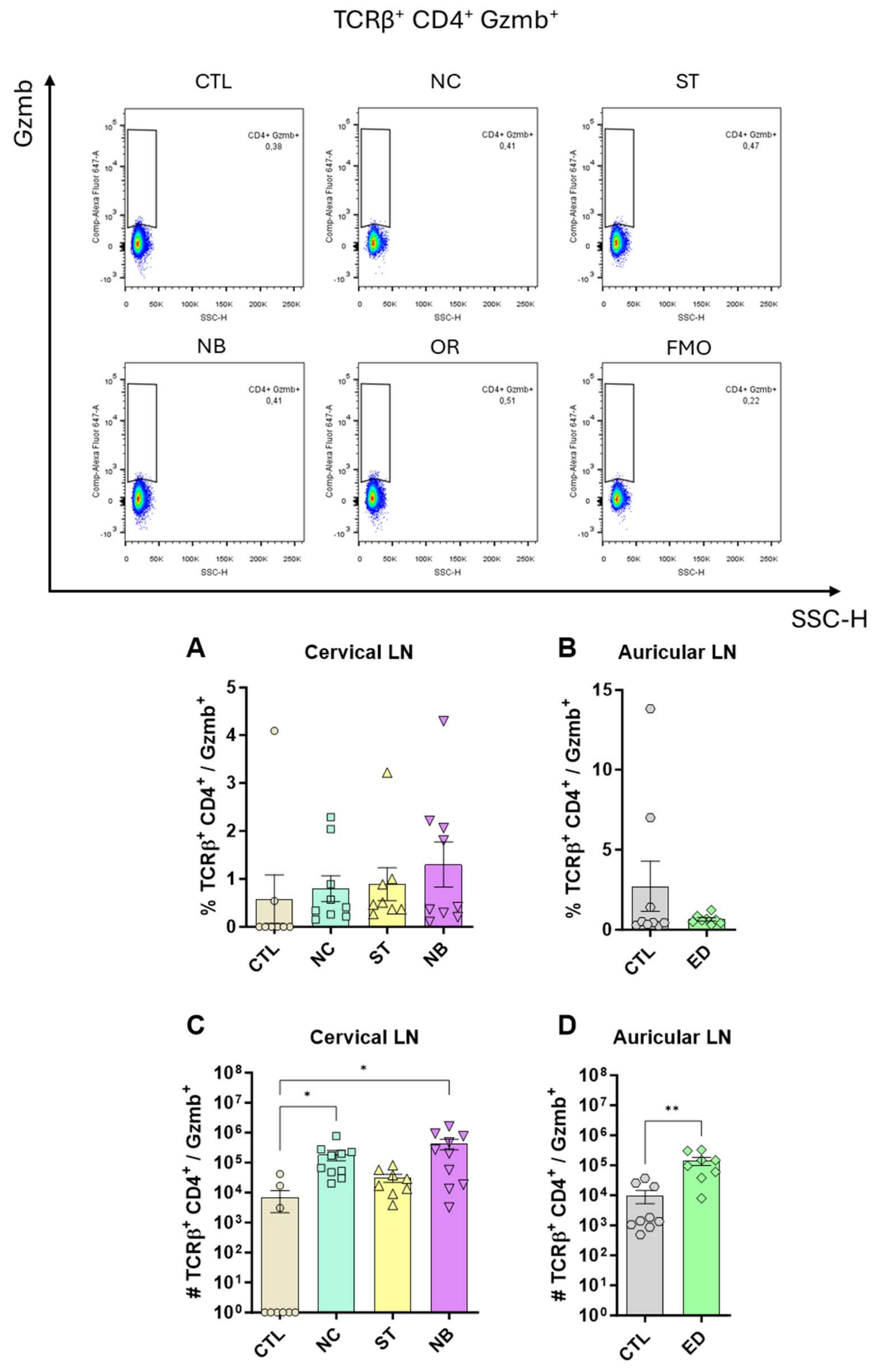

Figure S7. Profile of TCRβ⁺ CD4⁺ Gzmb⁺ Cells. The figure shows the representatives and graphs with the percentage of cells in the nasal and ear sites (A) and (B) respectively. In (C) and (D) data on the total number of cells are represented. Data accumulative of two independent

experiments. Statistics: plot with Standard Error of The Mean (SEM), t-test was used for all groups and samples * p<0,05, **<0,005., 3-6 animals per group in each.

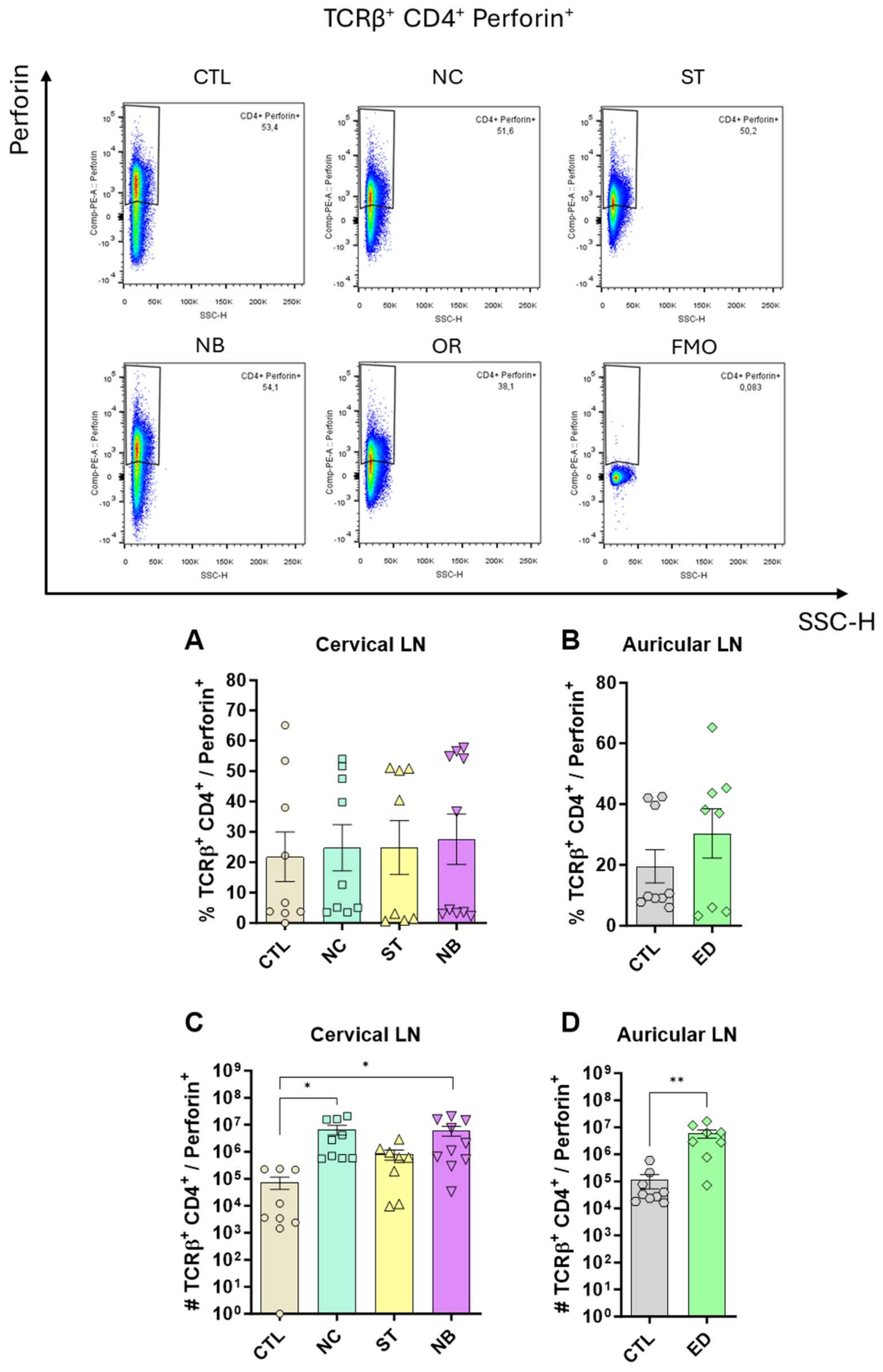

Figure S8. Profile of TCRβ^{+} CD4^{+} Perforin^{+} Cells. The figure shows the representatives and graphs with the percentage of cells in the nasal and ear sites (A) and (B) respectively. In (C) and (D) data on the total number of cells are represented. Data accumulative of two independent experiments. Statistics: plot with Standard Error of The Mean (SEM), t-test was used for all groups and samples * p<0,05, **<0,005., 3-6 animals per group in each.

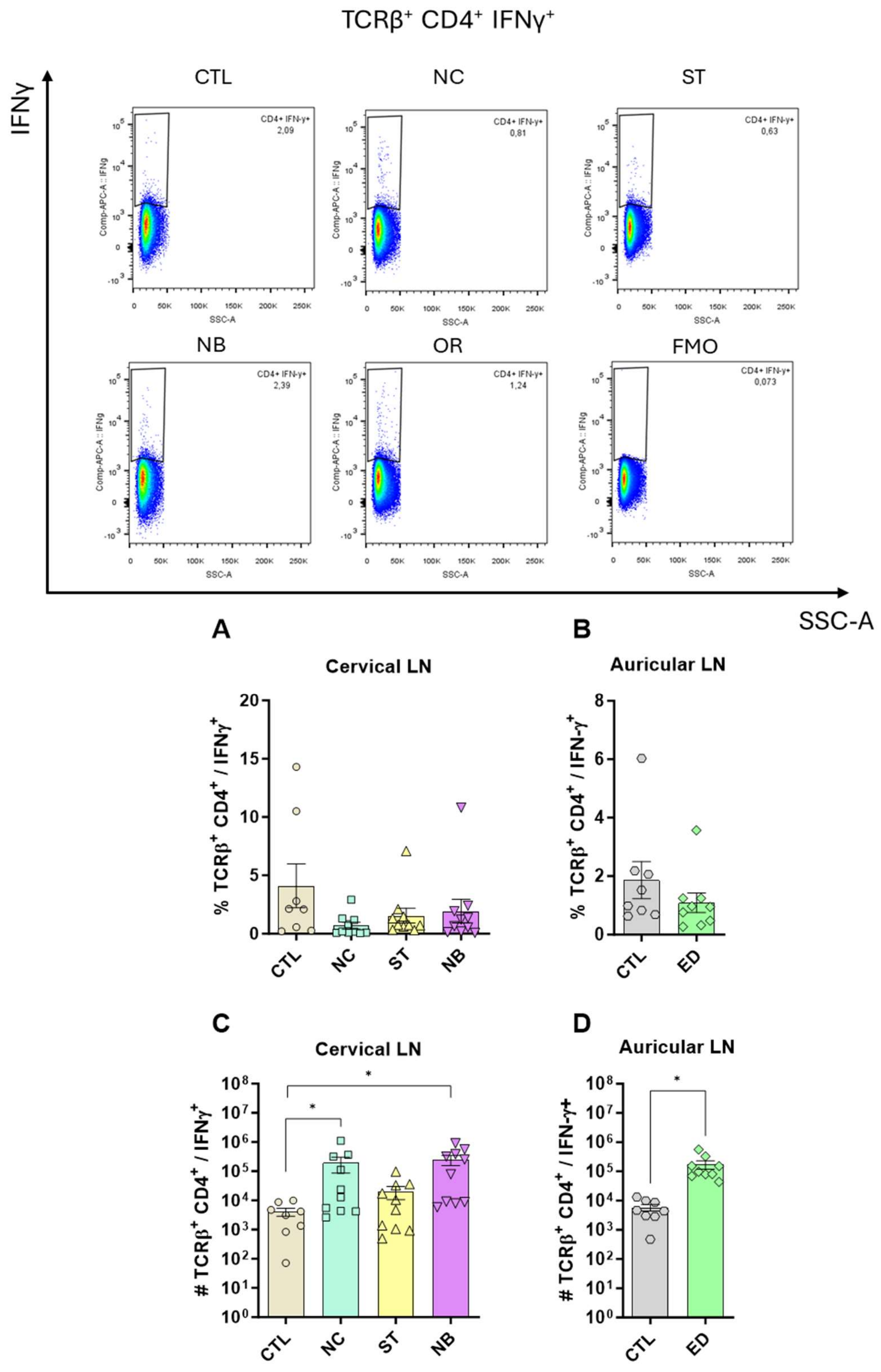

Figure S9. Profile of TCRβ⁺ CD4⁺ IFN-γ⁺ Cells. The figure shows the representatives and graphs with the percentage of cells in the nasal and ear sites (A) and (B) respectively. In (C) and (D) data on the total number of cells are represented. Data accumulative of two independent experiments.

Statistics: plot with Standard Error of The Mean (SEM), t-test was used for all groups and samples

* p<0,05, **<0,005., 3-6 animals per group in each.

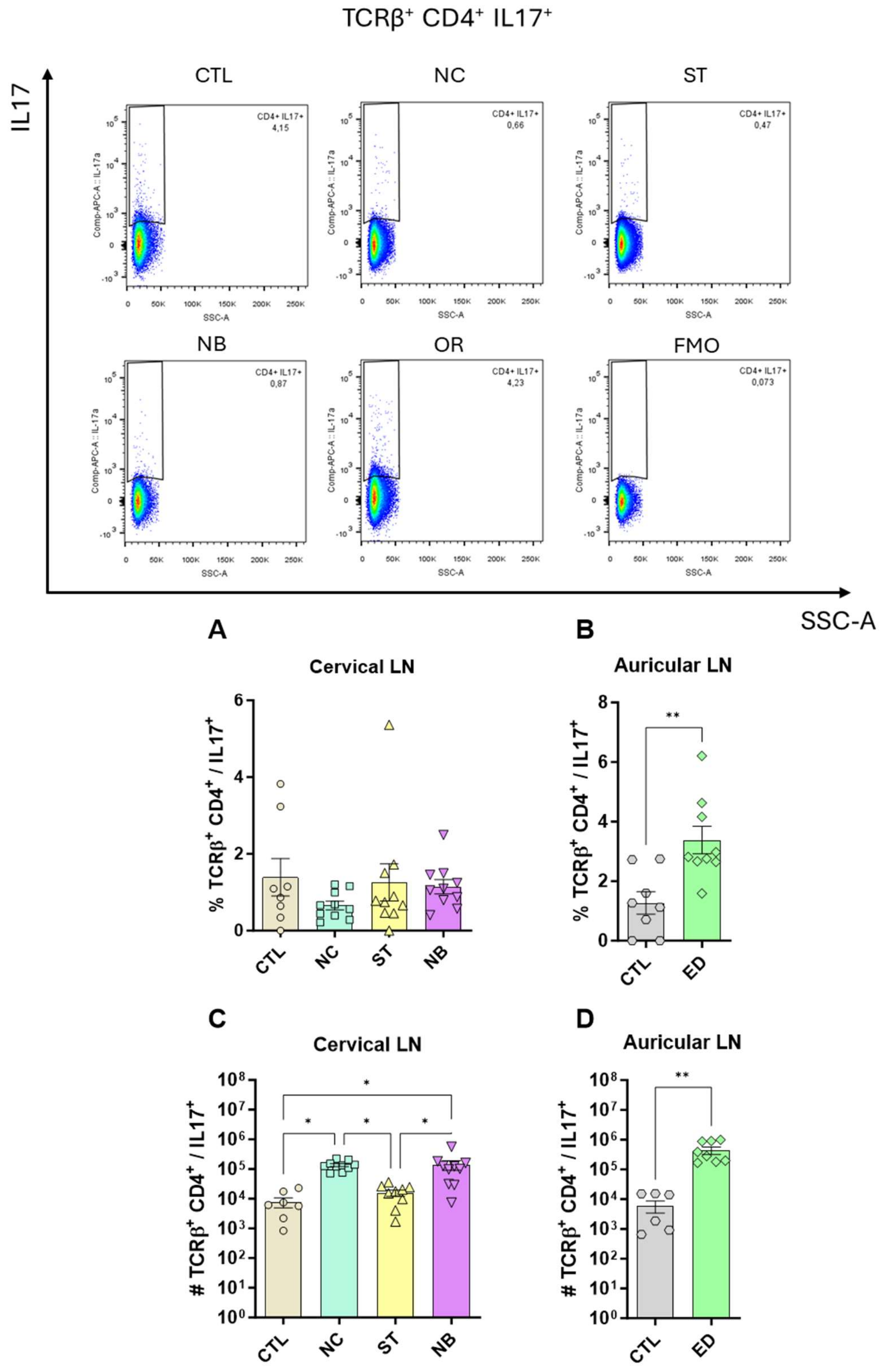

Figure S10. Profile of TCRβ⁺ CD4⁺ IL-17⁺ Cells. The figure shows the representatives and graphs with the percentage of cells in the nasal and ear sites (A) and (B) respectively. In (C) and (D) data on the total number of cells are represented. Data accumulative of two independent experiments. Statistics: plot with Standard Error of The Mean (SEM), t-test was used for all groups and samples * p<0,05, **<0,005., 3-6 animals per group in each.

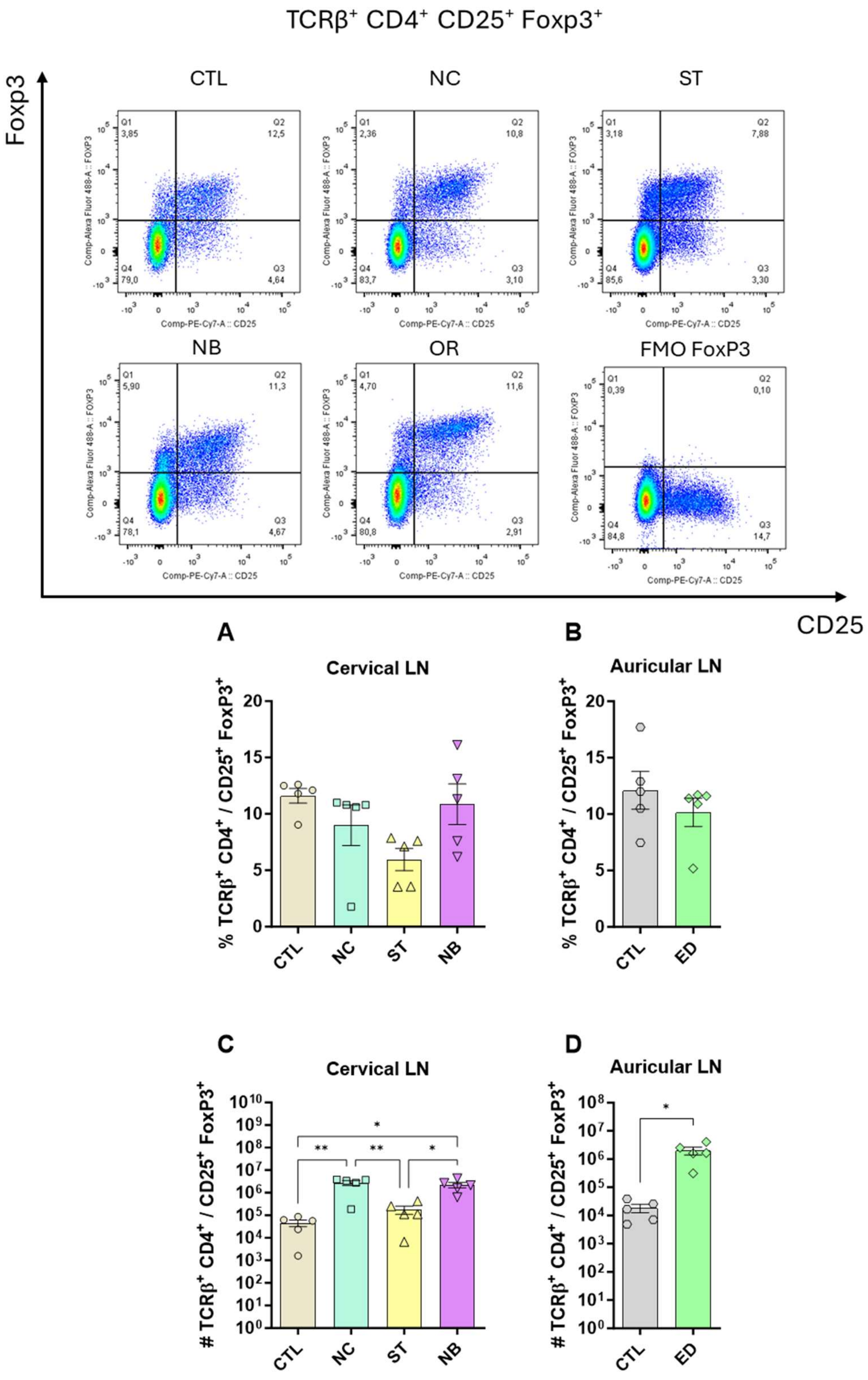

Figure S11. TCRβ$^+$ CD4$^+$ CD25$^-$ FoxP3$^+$ profile. The figure shows the representatives and graphs with the percentage of cells in the nasal and ear sites (A) and (B) respectively. In (C) and (D) data on the total number of cells are represented. Data accumulative of two independent

experiments. Statistics: plot with Standard Error of The Mean (SEM), t-test was used for all groups and samples * p<0,05, **<0,005., 3-6 animals per group in each.

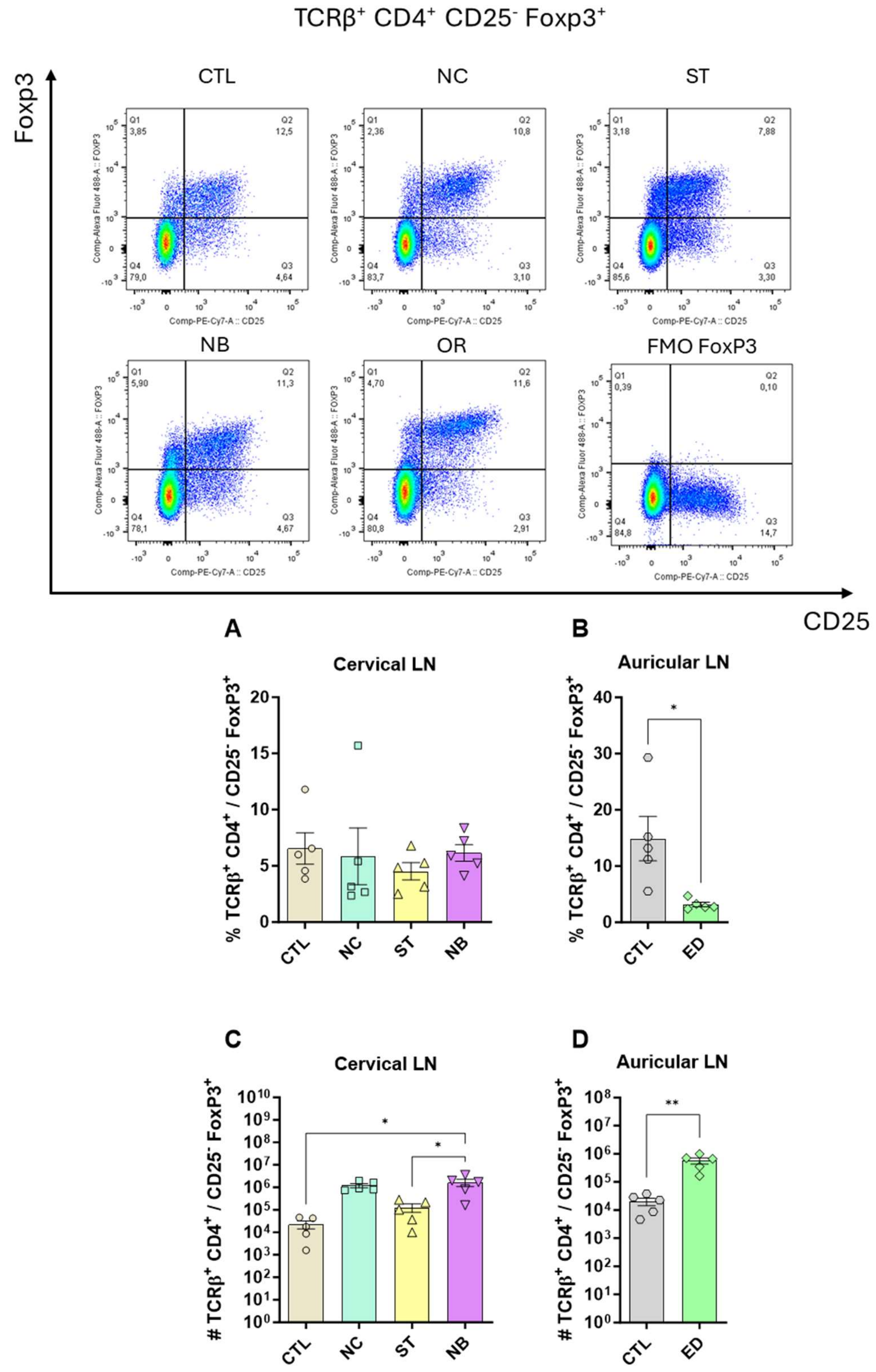

Figure S12. TCRβ$^+$ CD4$^+$ CD25$^-$ FoxP3$^+$ profile. The figure shows the representatives and graphs with the percentage of cells in the nasal and ear sites (A) and (B) respectively. In (C) and (D) data on the total number of cells are represented. Data of one independent experiment. Statistics: plot with Standard Error of The Mean (SEM), t-test was used for all groups and samples * p<0,05, **<0,005., 3-6 animals per group in each.

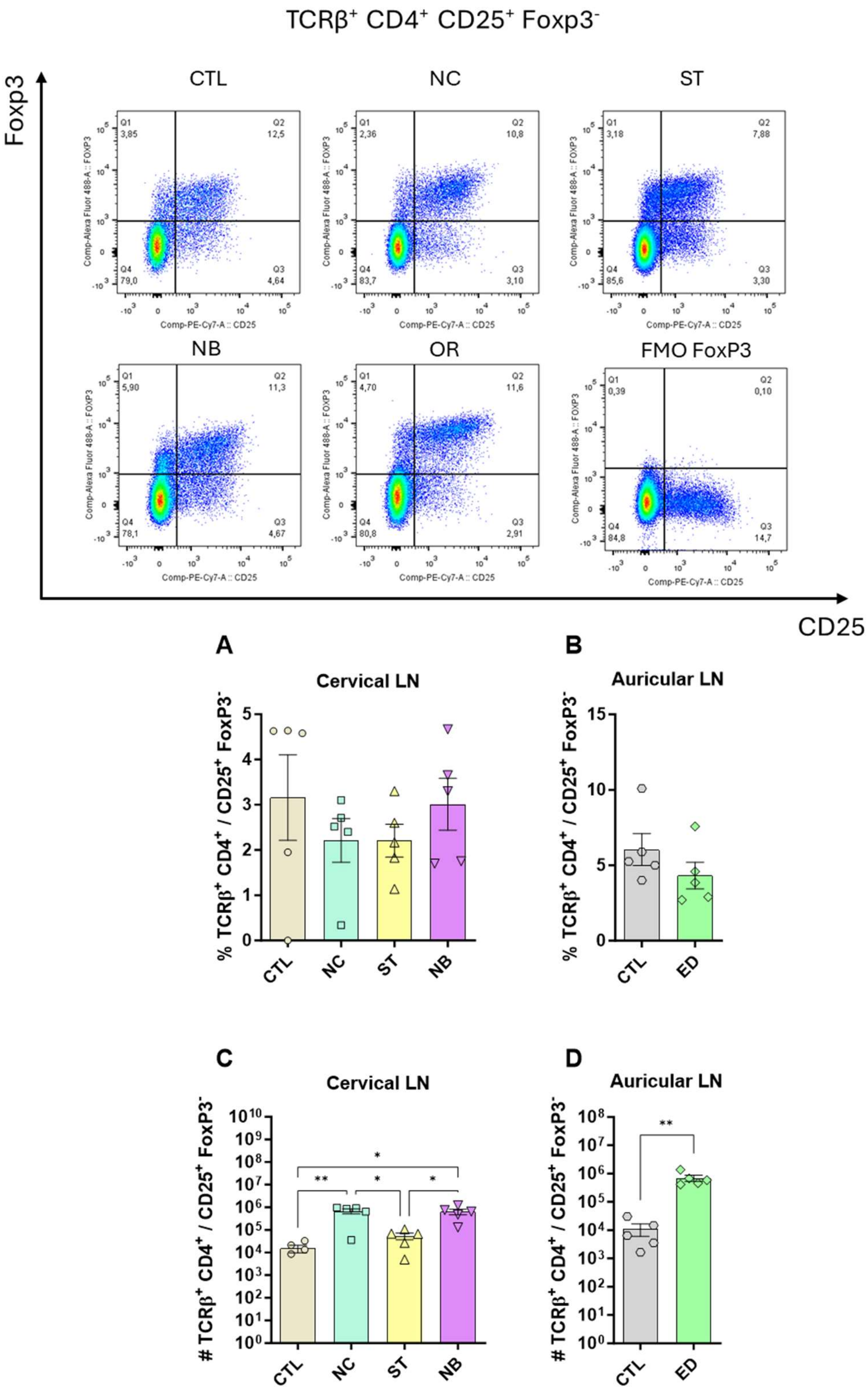

Figure S13. TCRβ⁺ CD4⁺ CD25⁺ FoxP3⁻ profile. The figure shows the representatives and graphs with the percentage of cells in the nasal and ear sites (A) and (B) respectively. In (C) and (D) data on the total number of cells are represented. Data of one independent experiment.

Statistics: plot with Standard Error of The Mean (SEM), t-test was used for all groups and samples

* p<0,05, **<0,005., 3-6 animals per group in each.

TCRβ⁺ CD4⁺ CD25⁻ Foxp3⁻

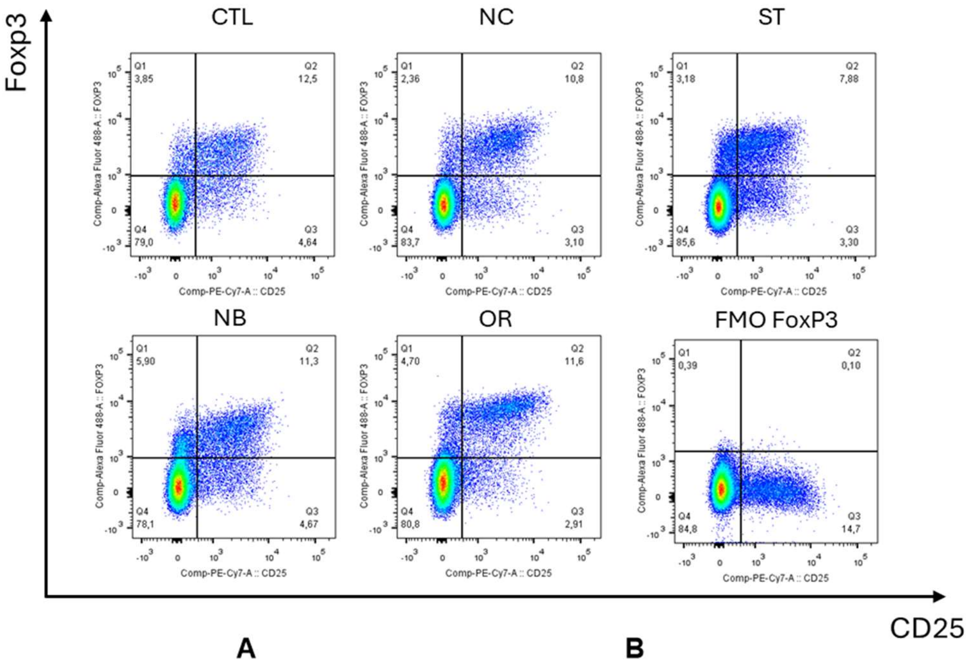

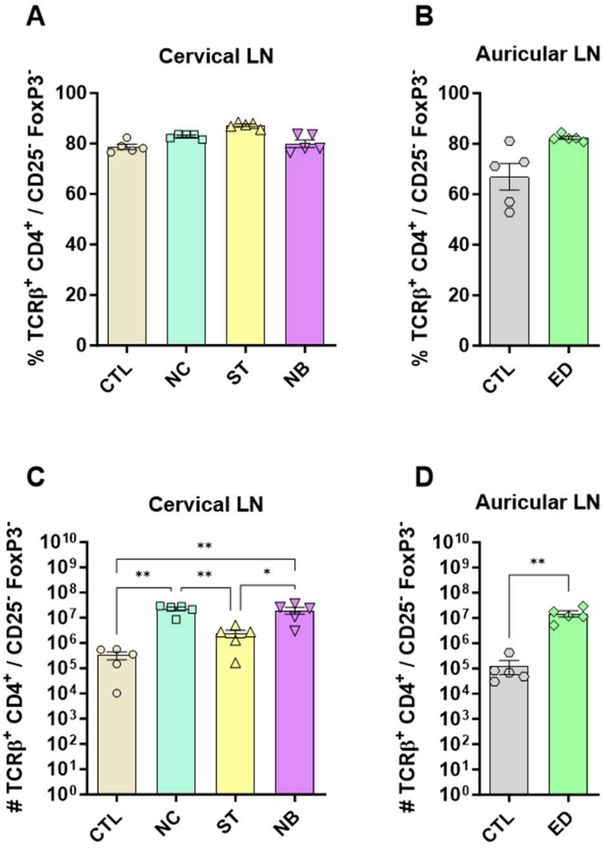

Figure S14. TCRβ$^+$ CD4$^+$ CD25$^-$ FoxP3$^-$ profile. The figure shows the representatives and graphs with the percentage of cells in the nasal and ear sites (A) and (B) respectively. In (C) and (D) data on the total number of cells are represented. Data of one independent experiment. Statistics: plot with Standard Error of The Mean (SEM), t-test was used for all groups and samples * p<0,05, **<0,005., 3-6 animals per group in each.

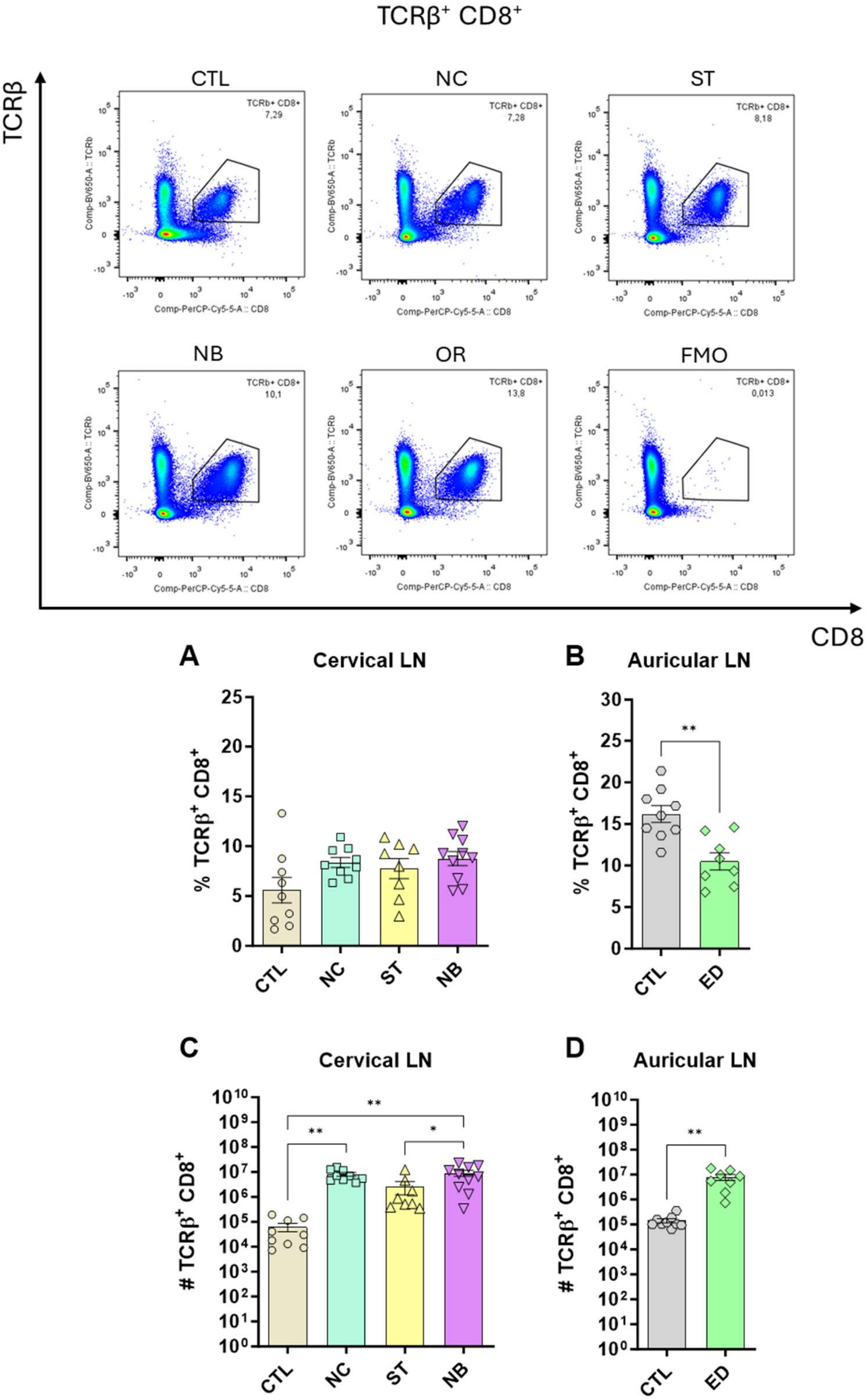

Figure S15. Profile of TCRβ⁺ CD8⁺ Cells. The figure shows the representatives and graphs with the percentage of cells in the nasal and ear sites (A) and (B) respectively. In (C) and (D) data on the total number of cells are represented. Data accumulative of two independent experiments.

Statistics: plot with Standard Error of The Mean (SEM), t-test was used for all groups and samples

* p<0,05, **<0,005., 3-6 animals per group in each.

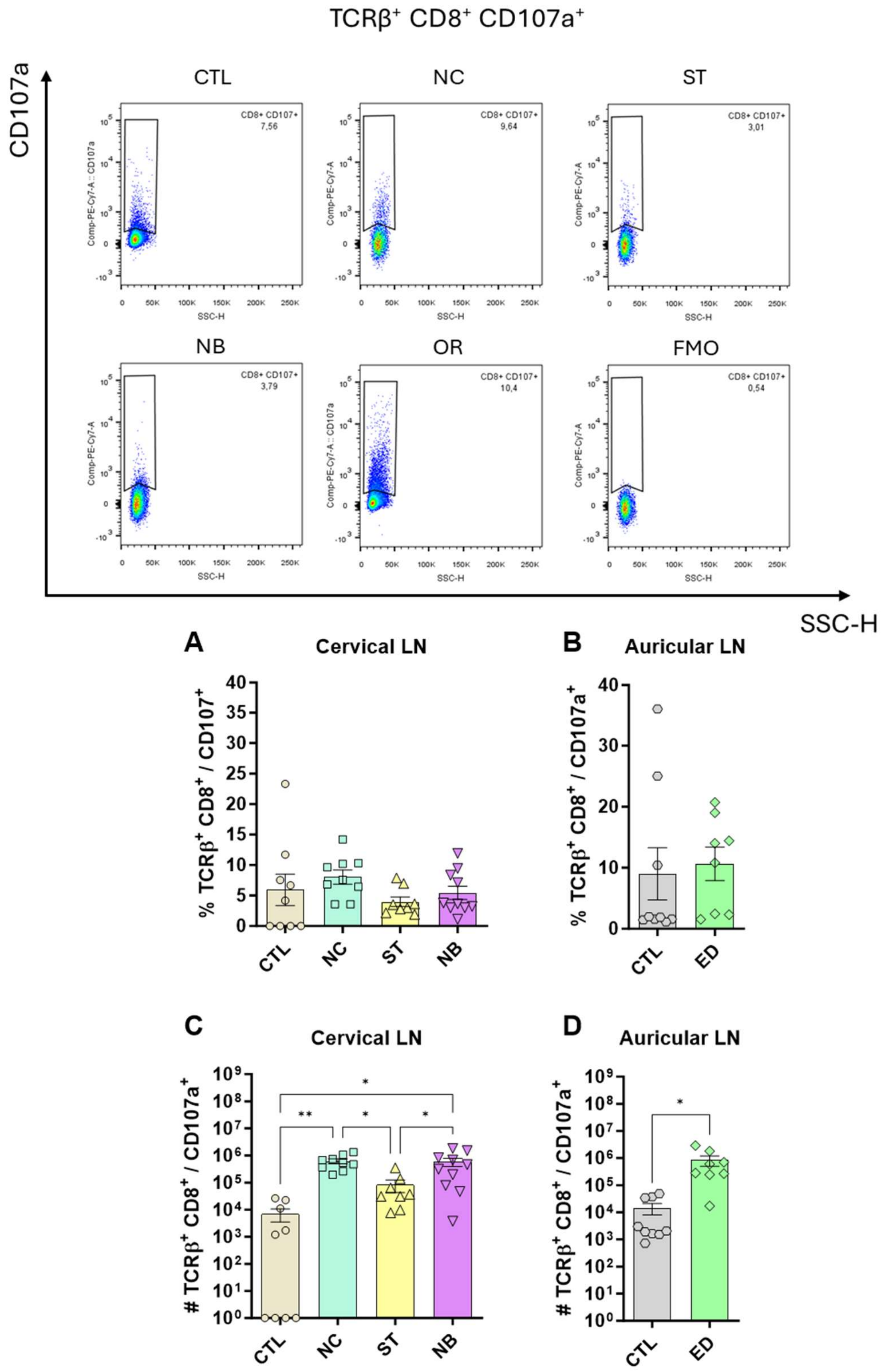

Figure S16. Profile of TCRβ$^+$ CD8$^+$ CD107a$^+$ Cells. The figure shows the representatives and graphs with the percentage of cells in the nasal and ear sites (A) and (B) respectively. In (C) and (D) data on the total number of cells are represented. Data accumulative of two independent experiments. Statistics: plot with Standard Error of The Mean (SEM), t-test was used for all groups and samples * p<0,05, **<0,005., 3-6 animals per group in each.

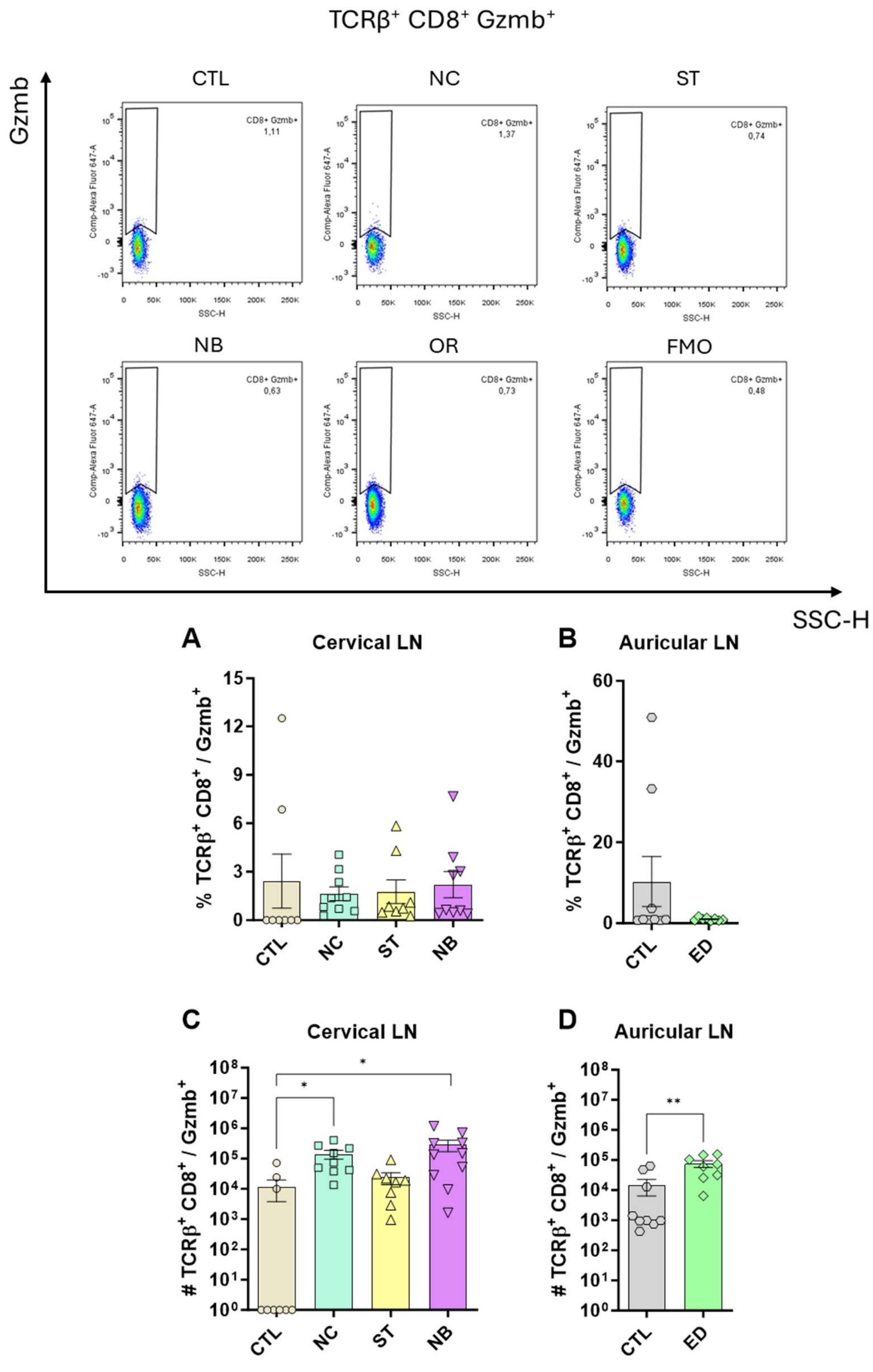

Figure S17. Profile of TCRβ⁺ CD8⁺ Gzmb⁺ Cells. The figure shows the representatives and graphs with the percentage of cells in the nasal and ear sites (A) and (B) respectively. In (C) and (D) data on the total number of cells are represented. Data accumulative of two independent

experiments. Statistics: plot with Standard Error of The Mean (SEM), t-test was used for all groups and samples * p<0,05, **<0,005., 3-6 animals per group in each.

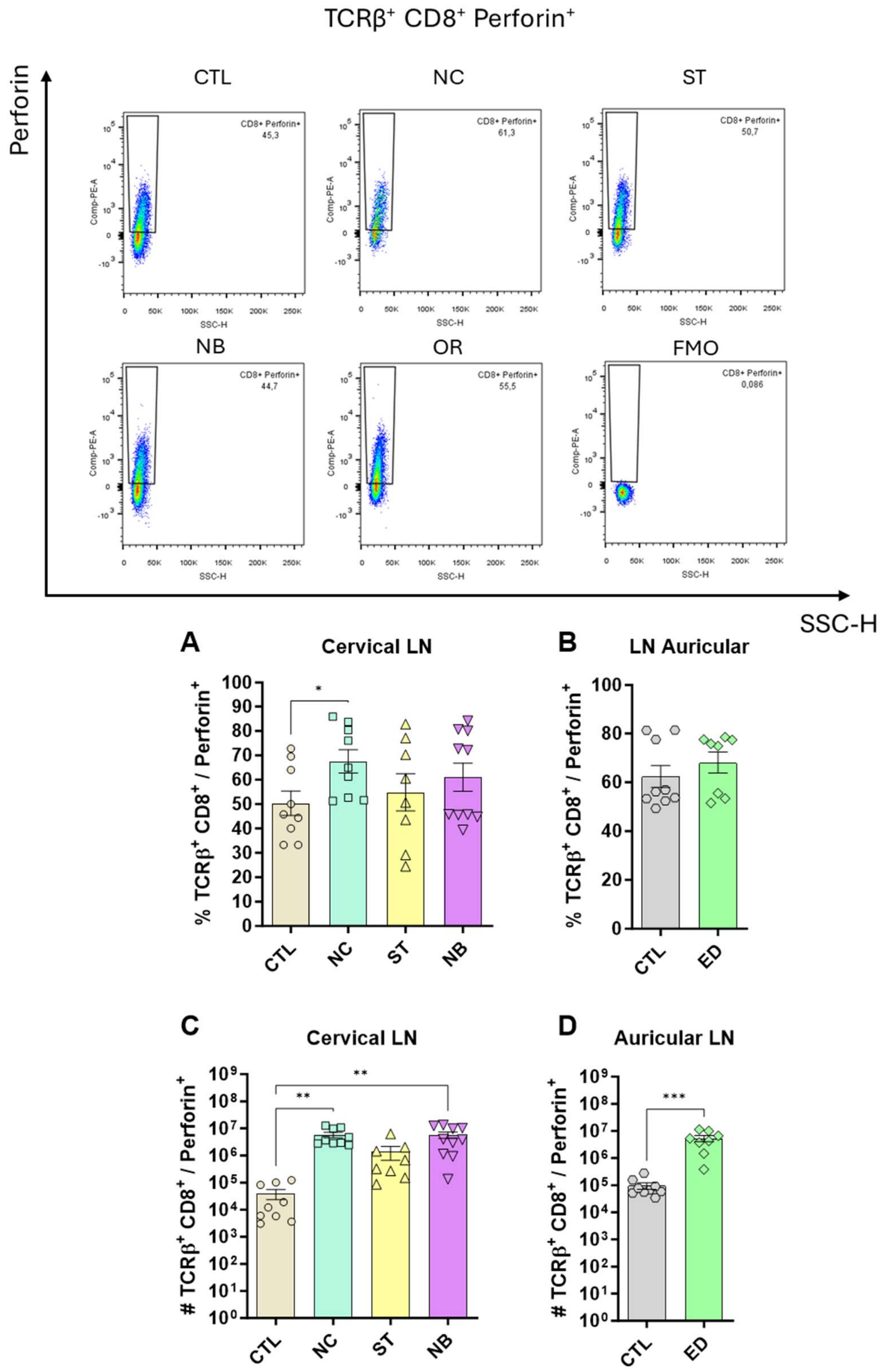

Figure S18. Profile of TCRβ^{+} CD8^{+} Perforin^{+} Cells. The figure shows the representatives and graphs with the percentage of cells in the nasal and ear sites (A) and (B) respectively. In (C) and (D) data on the total number of cells are represented. Data accumulative of two independent experiments. Statistics: plot with Standard Error of The Mean (SEM), t-test was used for all groups and samples * p<0,05, **<0,005., 3-6 animals per group in each.

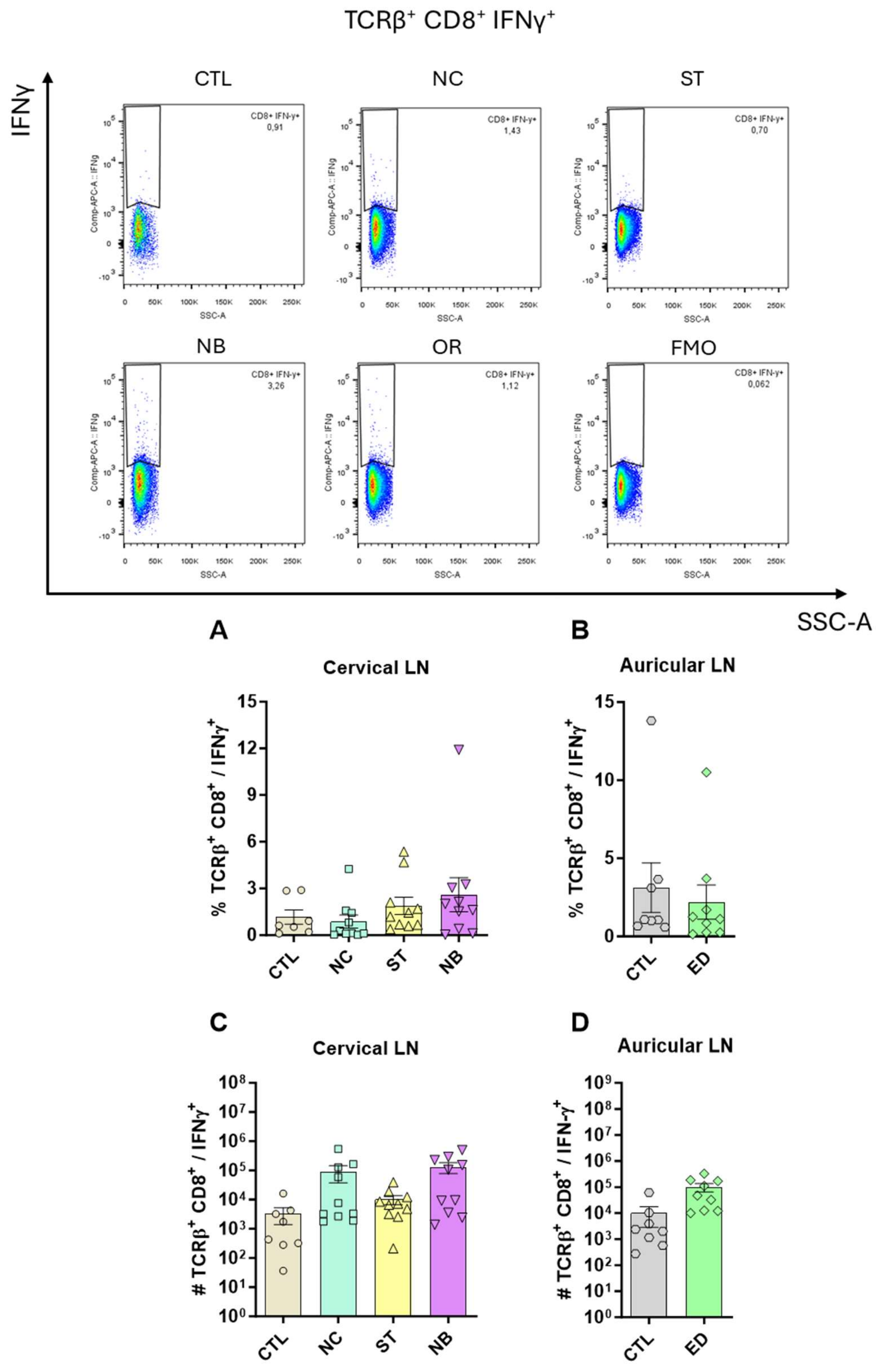

Figure S19. Profile of TCRβ⁺ CD8⁺ IFN-γ⁺ Cells. The figure shows the representatives and graphs with the percentage of cells in the nasal and ear sites (A) and (B) respectively. In (C) and (D) data on the total number of cells are represented. Data accumulative of two independent

experiments. Statistics: plot with Standard Error of The Mean (SEM), t-test was used for all groups and samples * p<0,05, **<0,005., 3-6 animals per group in each.

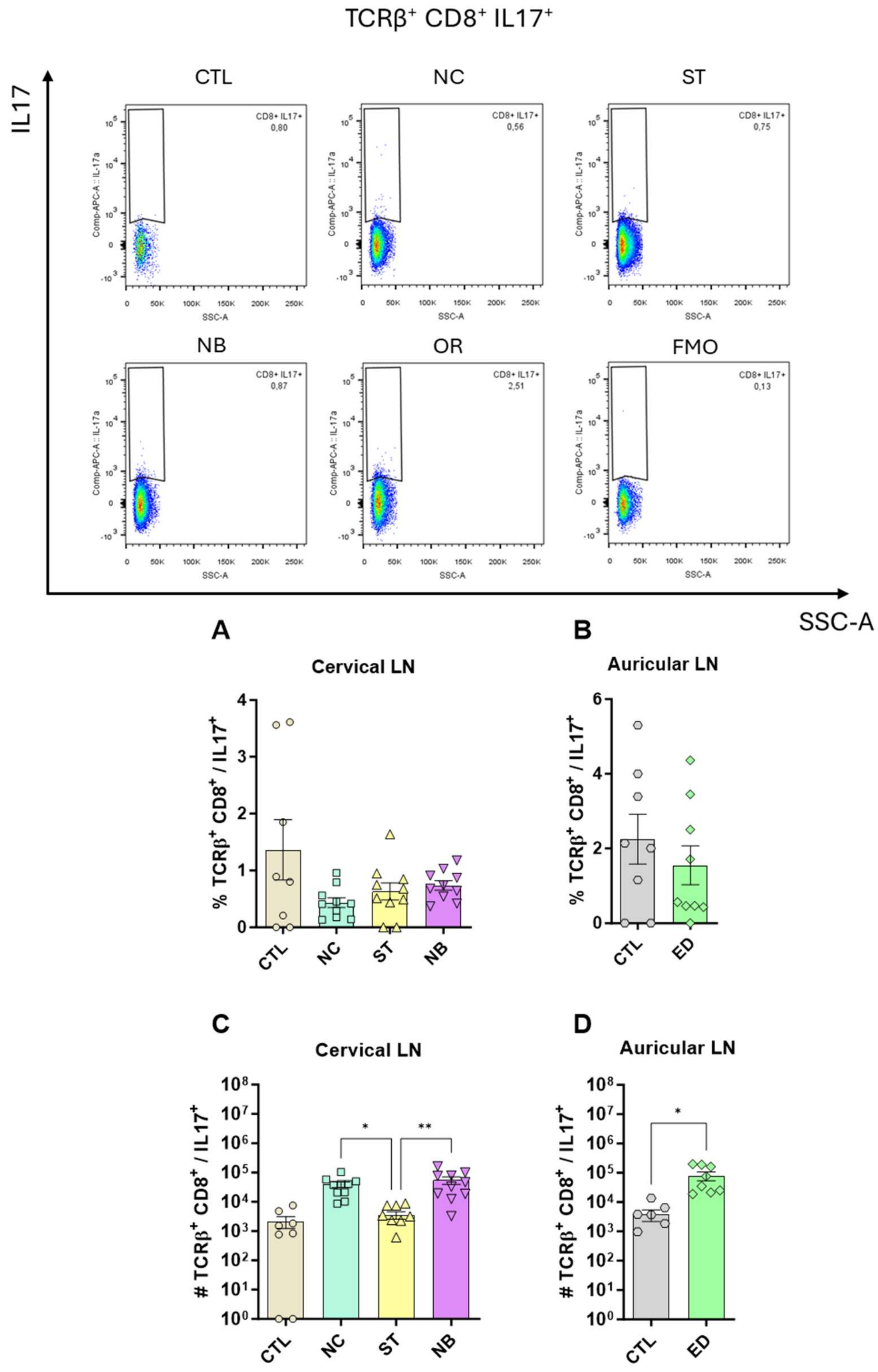

Figure S20. Profile of TCRβ^{+} CD8^{+} IL-17^{+} Cells. The figure shows the representatives and graphs with the percentage of cells in the nasal and ear sites (A) and (B) respectively. In (C) and (D) data on the total number of cells are represented. Data accumulative of two independent experiments. Statistics: plot with Standard Error of The Mean (SEM), t-test was used for all groups and samples * $p<0{,}05$, **<0,005., 3-6 animals per group in each.

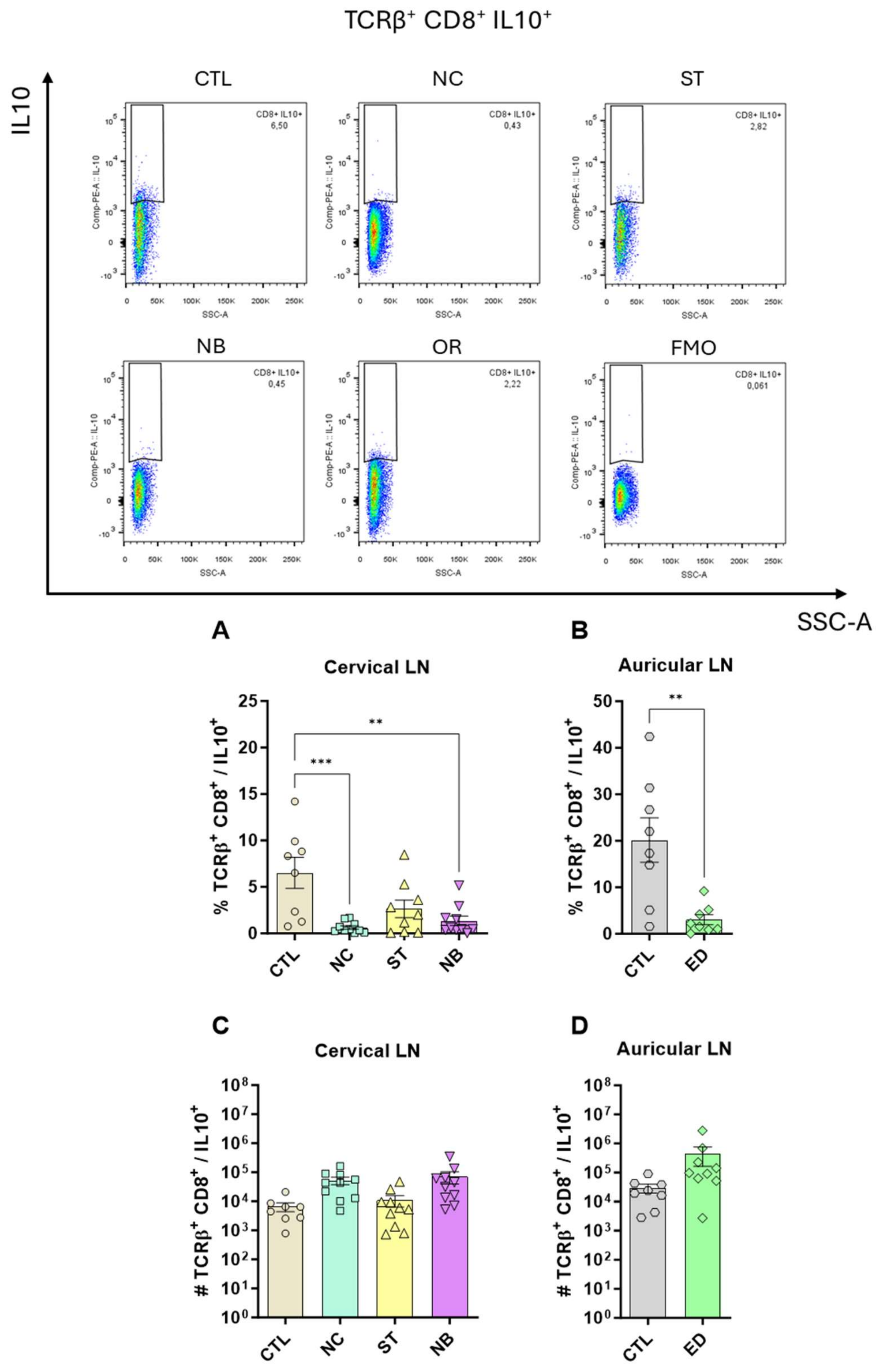

Figure S21. Profile of TCRβ⁺ CD8⁺ IL-10⁺ Cells. The figure shows the representatives and graphs with the percentage of cells in the nasal and ear sites (A) and (B) respectively. In (C) and (D) data on the total number of cells are represented. Data accumulative of two independent

experiments. Statistics: plot with Standard Error of The Mean (SEM), t-test was used for all groups and samples * p<0,05, **<0,005., 3-6 animals per group in each.

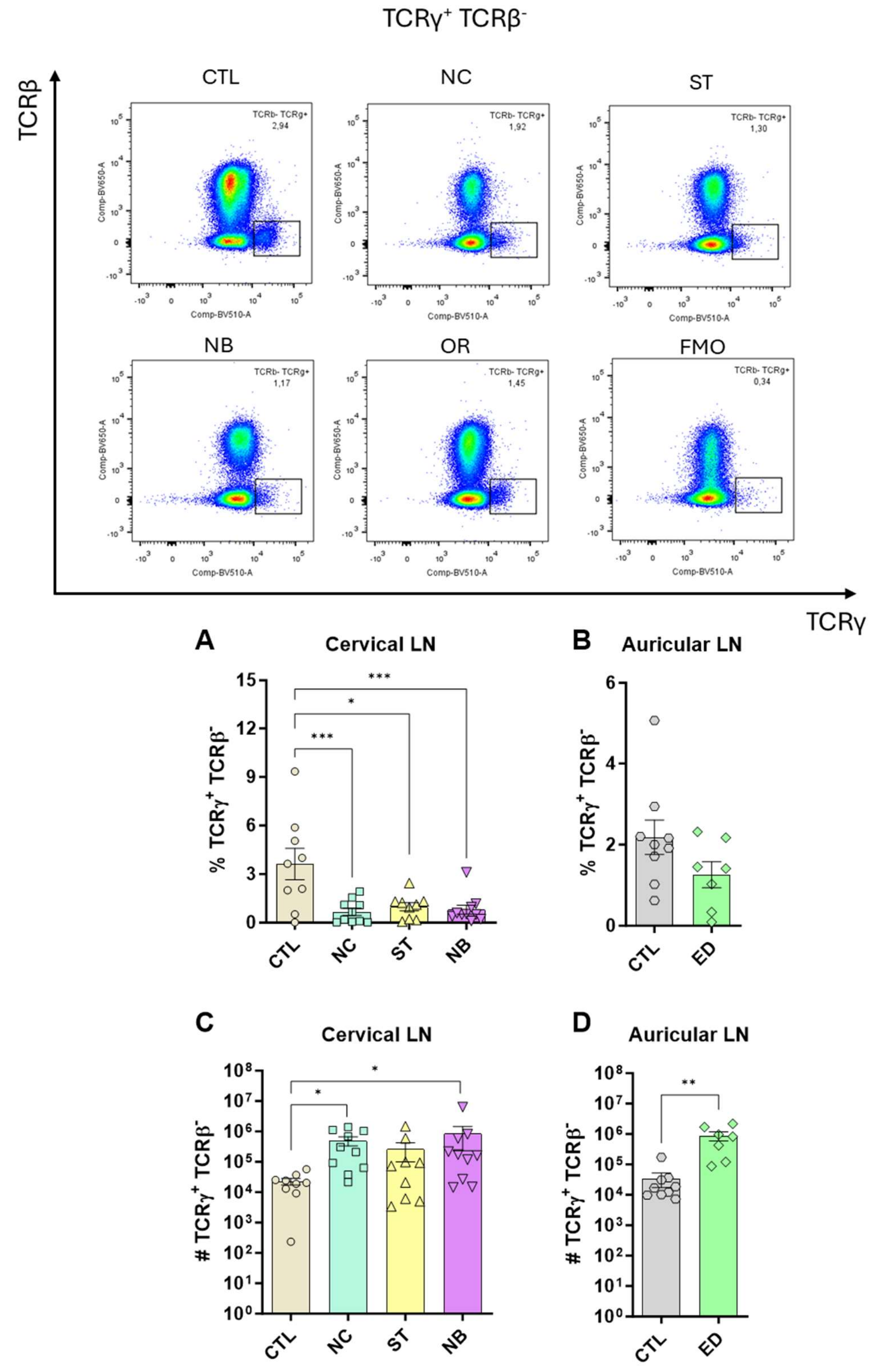

Figure S22. Profile of TCRγ⁺ TCRβ⁻ Cells. The figure shows the representatives and graphs with the percentage of cells in the nasal and ear sites (A) and (B) respectively. In (C) and (D) data on the total number of cells are represented. Data accumulative of two independent experiments. Statistics: plot with Standard Error of The Mean (SEM), t-test was used for all groups and samples * $p<0,05$, **<0,005., 3-6 animals per group in each.

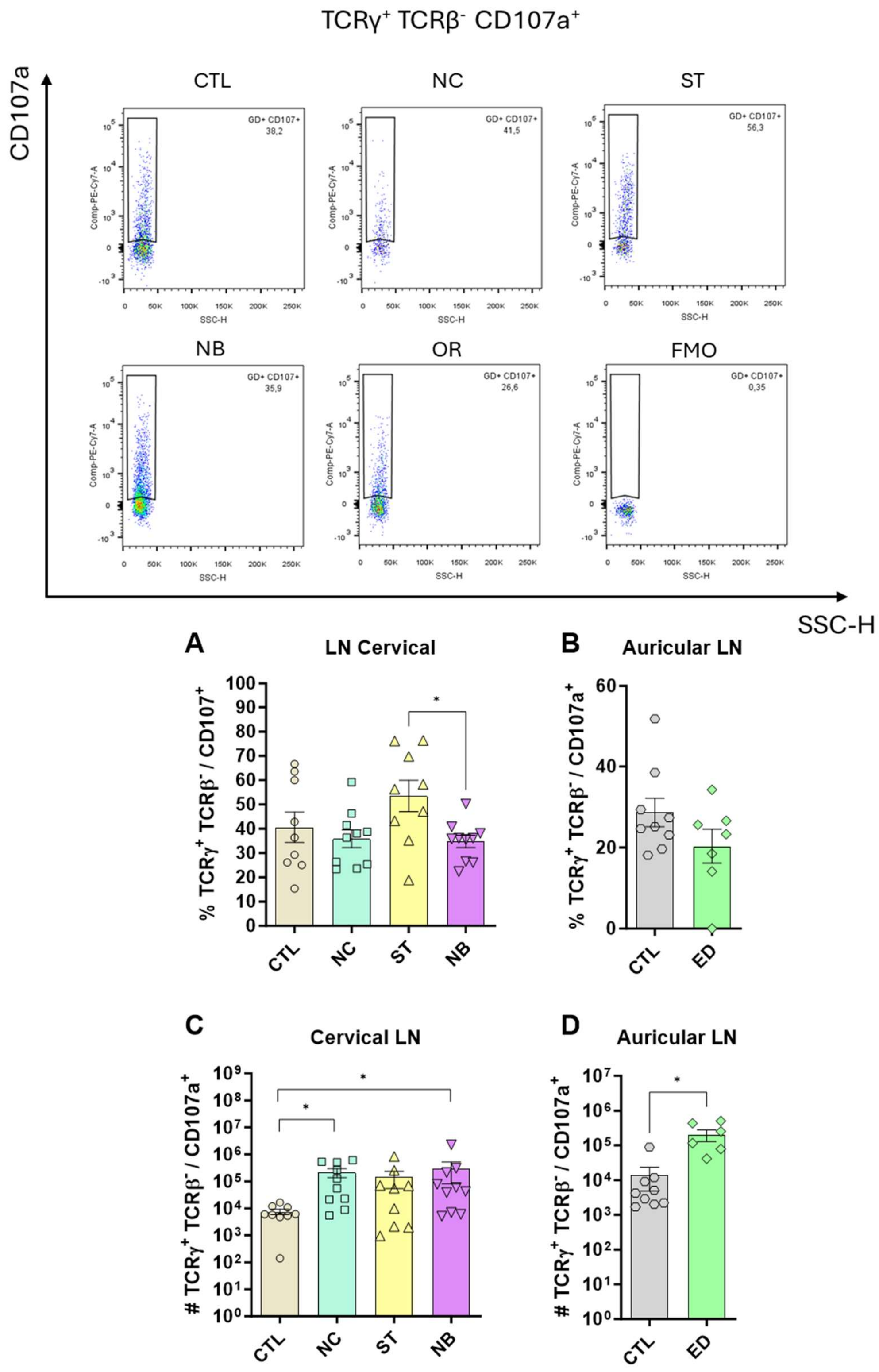

Figure S23. TCRγ⁺ TCRβ⁻ CD107a⁺ profile. The figure shows the representatives and graphs with the percentage of cells in the nasal and ear sites (A) and (B) respectively. In (C) and (D) data on the total number of cells are represented. Data accumulative of two independent experiments.

Statistics: plot with Standard Error of The Mean (SEM), t-test was used for all groups and samples

* p<0,05, **<0,005., 3-6 animals per group in each.

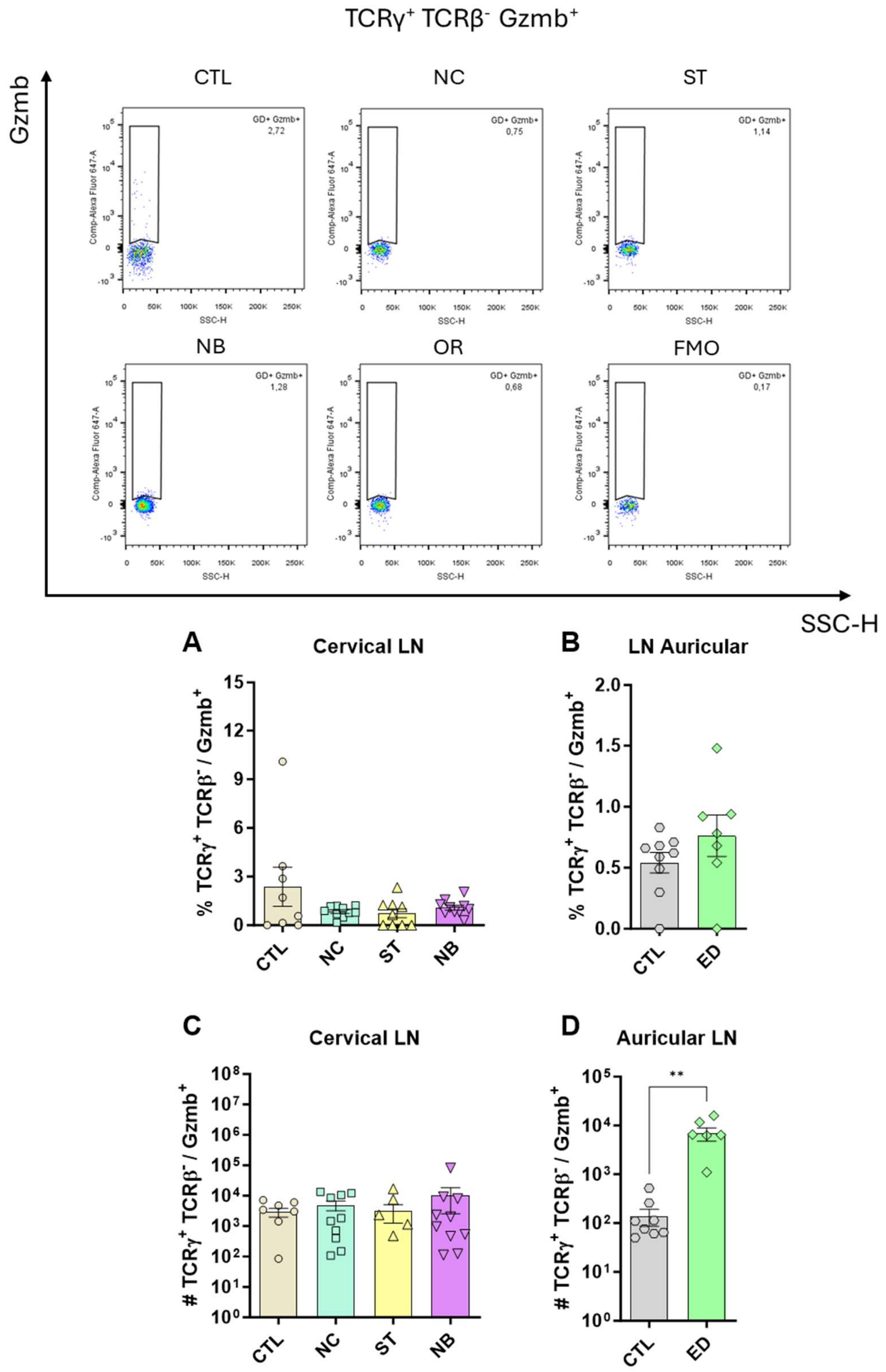

Figure S24. TCRγ^{+} TCRβ^{-} Gzmb^{+} profile. The figure shows the representatives and graphs with the percentage of cells in the nasal and ear sites (A) and (B) respectively. In (C) and (D) data on the total number of cells are represented. Data accumulative of two independent experiments. Statistics: plot with Standard Error of The Mean (SEM), t-test was used for all groups and samples * p<0,05, **<0,005., 3-6 animals per group in each.

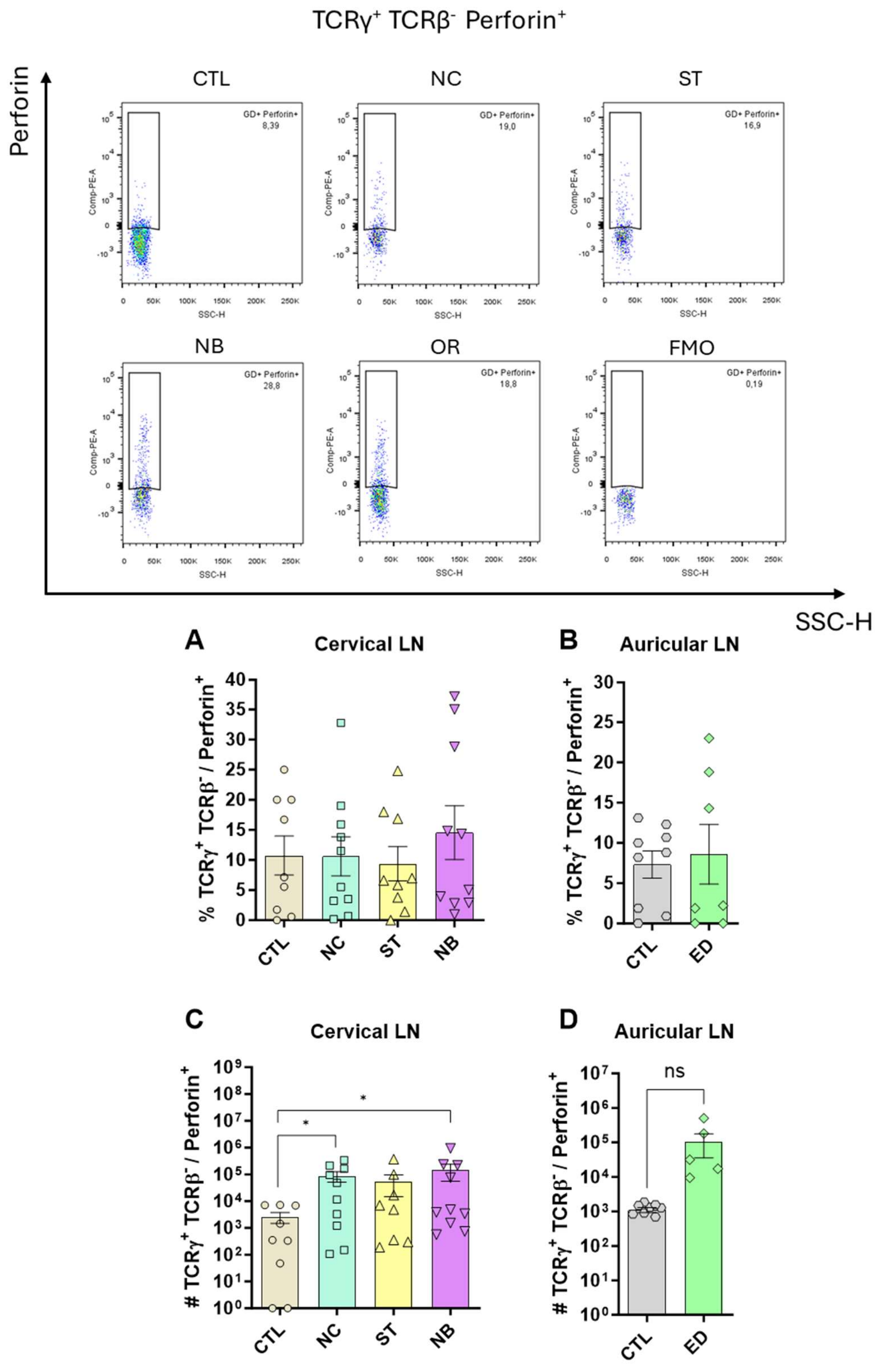

Figure S25. TCRγ⁺ TCRβ⁻ Perforin⁺ profile. The figure shows the representatives and graphs with the percentage of cells in the nasal and ear sites (A) and (B) respectively. In (C) and (D) data on the total number of cells are represented. Data accumulative of two independent experiments.

Statistics: plot with Standard Error of The Mean (SEM), t-test was used for all groups and samples

* p<0,05, **<0,005., 3-6 animals per group in each.

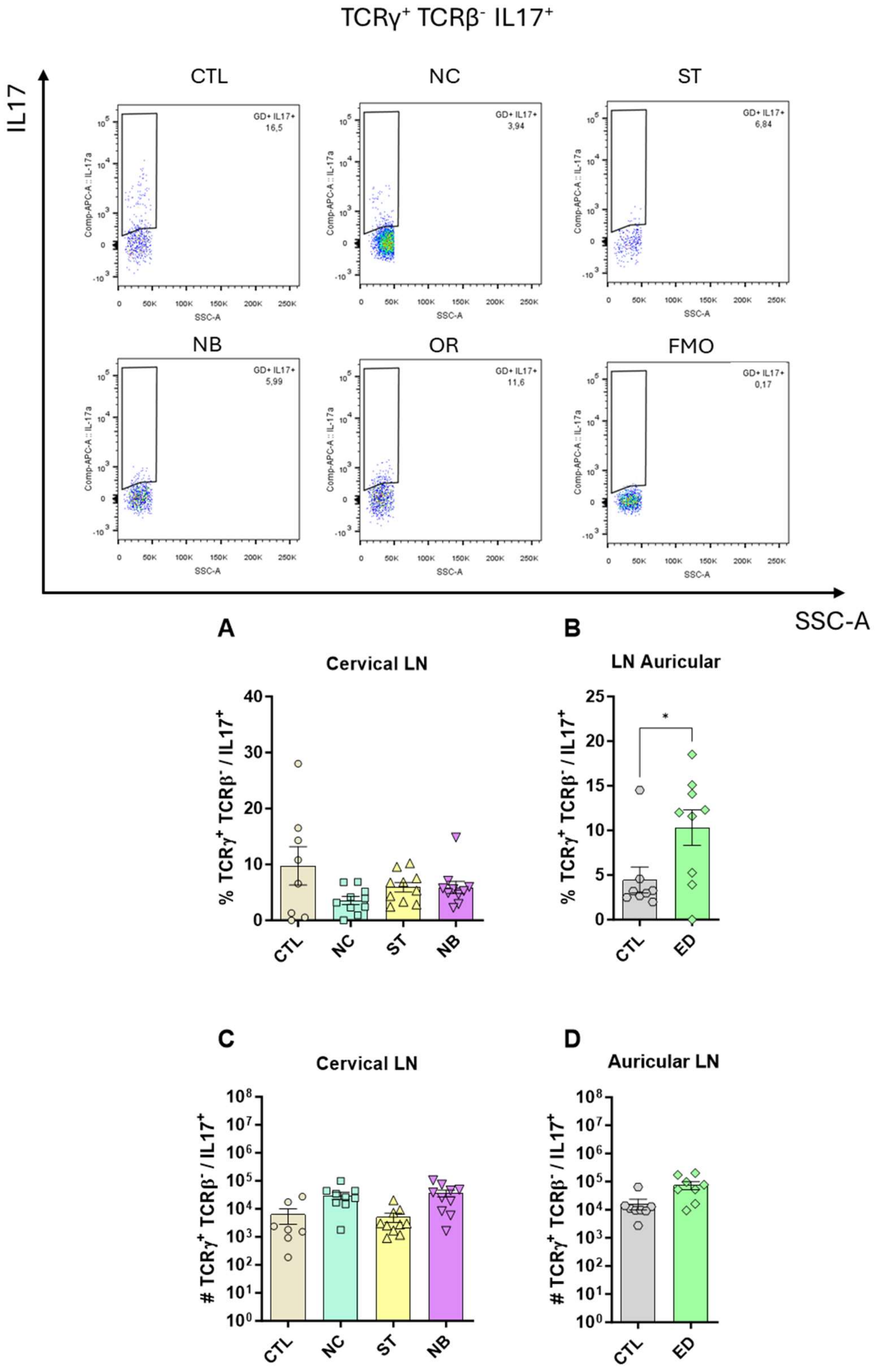

Figure S26. TCRγ^{+} TCRβ^{-} IL-17^{+} profile. The figure shows the representatives and graphs with the percentage of cells in the nasal and ear sites (A) and (B) respectively. In (C) and (D) data on the total number of cells are represented. Data accumulative of two independent experiments. Statistics: plot with Standard Error of The Mean (SEM), t-test was used for all groups and samples * p<0,05, **<0,005., 3-6 animals per group in each.

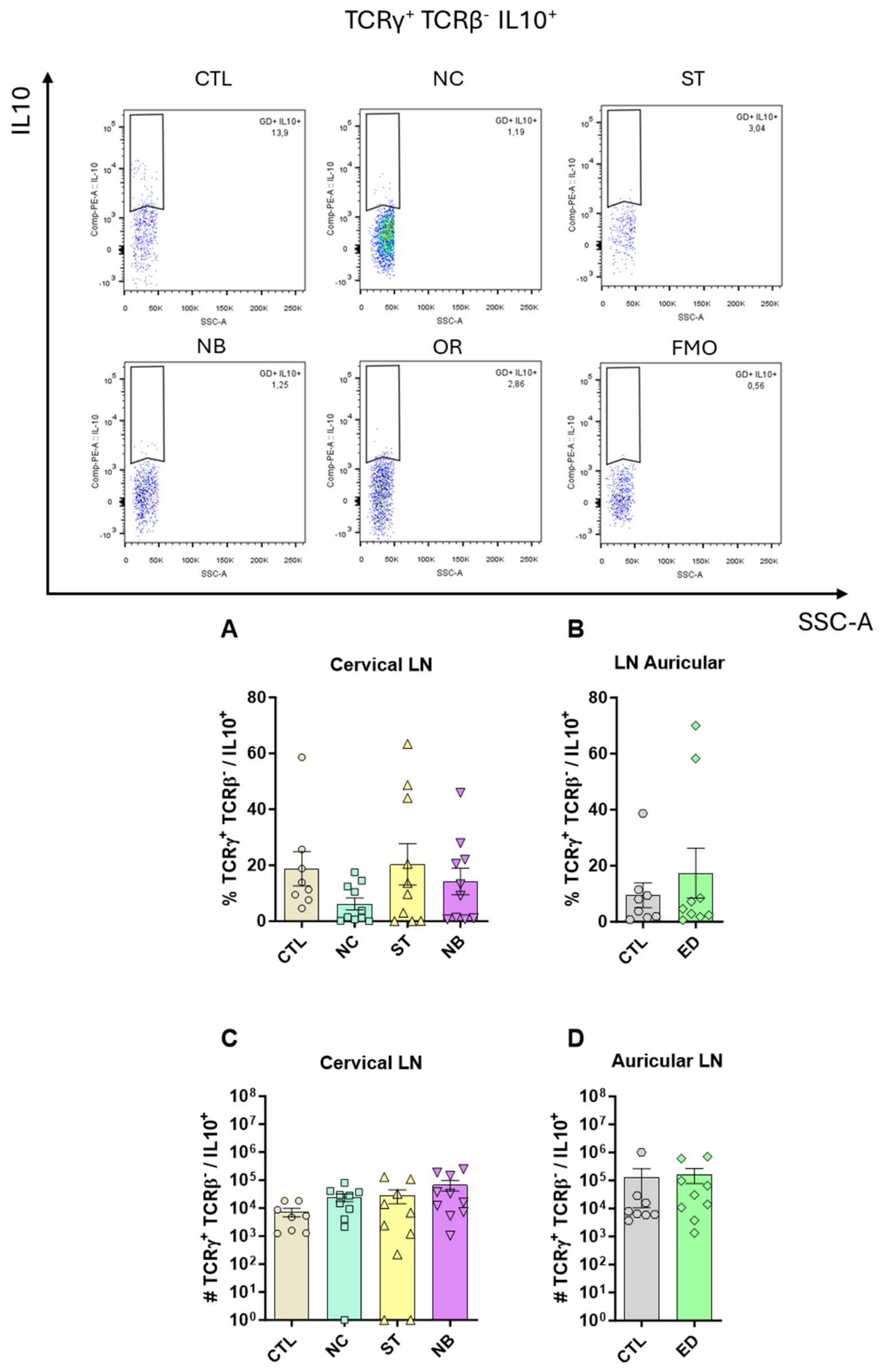

Figure S27. TCRγ^{+} TCRβ^{-} IL-10^{+} profile. The figure shows the representatives and graphs with the percentage of cells in the nasal and ear sites (A) and (B) respectively. In (C) and (D) data on the total number of cells are represented. Data accumulative of two independent experiments.

Statistics: plot with Standard Error of The Mean (SEM), t-test was used for all groups and samples

* p<0,05, **<0,005., 3-6 animals per group in each.

Table S1. BD LSR Fortessa X20 Cytometer Lasers and Filters Configuration.

Laser	Filter BP	Filter LP	Laser	Filter BP	Filter LP
**Violet**	780/60<br>710/50<br>670/30<br>610/20<br>525/50<br>450/50	750<br>690<br>655<br>595<br>505<br>---	**Yellow-Green**	780/60<br>710/50<br>670/30<br>60/20<br>586/15	750<br>685<br>635<br>600<br>---
**Blue**	710/50<br>530/30	690<br>505	**Red**	780/60<br>730/45<br>670/30	750<br>710<br>---

Table S2. Main staining antibodies markers, colors and concentrations.

Extracellular			Intracellular		
**Antibody**	**Fluorocrome**	**Concentration**	**Antibody**	**Fluorocrome**	**Concentration**
anti-CD3	BV605	1μg/mL	anti-IL-10	PE	2μg/mL
anti-CD4	BV785	1μg/mL	anti-IL-17	APC	2μg/mL
anti-CD8	Percp Cy5.5	1μg/mL	anti-IFN-γ	FITC	2μg/mL
anti-TCRβ	BV650	1μg/mL	anti-Perforin	PE	2μg/mL
anti-TCRγ	BV510	1μg/mL	anti-Granzyme b	APC	2μg/mL
anti-CD25	PE Cy7	1μg/mL	anti-CD107a	APC Cy7	2μg/mL
anti-PD-1	PE-Cy7	1μg/mL	anti-FoxP3	AF488	2μg/mL
